# Volumetric growth of soft tissues evaluated in the current configuration

**DOI:** 10.1007/s10237-021-01549-y

**Published:** 2022-01-19

**Authors:** X Zhuan, X Y Luo

**Affiliations:** School of Mathematics and Statistics, University of Glasgow, Glasgow, UK

**Keywords:** Soft tissues, Volumetric growth, Growth tensor, Growth law, Current configuration, Reference configuration, Myocardium, Residual stress

## Abstract

The growth and remodelling of soft tissues plays a significant role in many physiological applications, particularly in understanding and managing many diseases. A commonly used approach for soft tissue growth and remodelling is volumetric growth theory, introduced in the framework of finite elasticity. In such an approach, the total deformation gradient tensor is decomposed so that the elastic and growth tensors can be studied separately. A critical element in this approach is to determine the growth tensor and its evolution with time. Most existing volumetric growth theories define the growth tensor in the reference (natural) configuration, which does not reflect the continuous adaptation processes of soft tissues under the current configuration. In a few studies where growth from a loaded configuration was considered, simplifying assumptions, such as compatible deformation or geometric symmetries, were introduced. In this work, we propose a new volumetric growth law that depends on fields evaluated in the current configuration, which is residually stressed and loaded, without any geometrical restrictions. We illustrate our idea using a simplified left ventricle model, which admits inhomogeneous growth in the current configuration. We compare the residual stress distribution of our approach with the traditional volumetric growth theory, that assumes growth occurring from the natural reference configuration. We show that the proposed framework leads to qualitative agreements with experimental measurements. Furthermore, using a cylindrical model, we find an incompatibility index that explains the differences between the two approaches in more depth. We also demonstrate that results from both approaches reach the same steady solution published previously at the limit of a saturated growth. Although we used a left ventricle model as an example, our theory is applicable in modelling the volumetric growth of general soft tissues.

## Introduction

The interactions between living organs and the bio-environment play essential roles in regulating pathological or physiological growth. It has been experimentally demonstrated that environmental factors, such as the chemical, mechanical or genetic stimulus, could induce *growth and remodelling* (G&R) processes in living organs. Living organs can re-shape themselves, reset their constituents’ growth (or turnover) rates, and develop volumetric and mass changes to adapt to pathological or physiological changes in the bio-environment. G&R has been observed in different forms. For instance, nutrition concentration will be increased around the tumours, which helps to accelerate cell division and lead to the local growth of tumours at the tissue level (chemical factors). Embryonic or young organs can continuously re-structure themselves to develop required functions. Mature organs are expected to stay in a relatively stable living state and serve as fully functional; however, pathologically, the dynamic impact of bio-environmental changes will induce a quick remodelling process to renovate the functional tissue. For instance, an embryo heart can continuously and instinctively develop its heart structure due to genetic factors, and tumours may grow independently of mechanical factors (Volokh 2006). However, a mature heart seems to grow along the direction of principal tensile stress due to mechanical factors (Taber and Eggers 1996). There are two typical examples of the maladaptive G&R in the heart. One is known as eccentric hypertrophy in response to chronic volume overload, in which increased diastole wall stress leads to the addition of sarcomeres in series, associated with ventricular dilation. The other is eccentric hypertrophy due to pressure overload, whereby an increased systolic wall stress leads to the addition of sarcomeres in parallel, resulting in wall-thickening.

Despite the importance of G&R in disease progression, the principles governing those mechanisms are not fully understood. Improving this field’s knowledge will have considerable potential for optimizing clinical treatments to save more lives efficiently. However, although growth has been of research interests for a few decades, and many different G&R theories exist but these mostly fall into two categories. The first one is based on variable material points, and the second one is based on fixed material points. The most well-known approach in the first category is known as the “constrained mixture” theory, initially developed by  Humphrey and Rajagopal (2002), in which living tissues are considered to be composed of different constituents which continuously turnover with mass changes during the life cycle. They postulate that each constituent has a “preferred state” and that the reference (natural) configurations for each constituent exist and will be whatever allows this preferred state to be achieved. This framework considers the reproduction and removal of each constituent, so the reference configurations can be different even for the same constituents. In essence, one can add or remove material points to change the reference configurations. Many researchers, e.g. Baek et al. (2006); Alford et al. (2008); Valentin and Humphrey (2009), and Watton et al. (2004, 2009); Watton and Hill (2009) developed their models in this category to study the G&R of arteries and aneurysms. The difficulty of using this theory is the lack of experimental data for historical changes of all the constituents during the tissue growth and it is almost impossible to track different natural configurations from experiments. Stimuli from combined effects of various sources, including mechanical, thermal, electrical, and genetic information, may simultaneously contribute to the successive growth processes in living organs over different time scales, raising the complexity of identifying the contributions of individual environmental factors.

The volumetric growth theory is a typical example of the second category, following the idea of the kinematics of multiplicative plasticity (Bilby et al. 1955; Kerckhoffs et al. 2012). In this theory, it is assumed that there exists one reference (natural) configuration for all the constituents, and any incompatible growth due to growth of different constituents can be absorbed by the residual stresses. Taber (1998) and Rachev et al. (1998),among others, have employed this concept to model arteries and their response to hypertension in maturity. Following a growth law introduced by Taber (1998); Kerckhoffs (2012) used a strain-driven law to model post-natal cardiac growth, in which a cumulative growth is considered using multiplicative decomposition of a consecutive sequence of growth and elastic deformation tensors. Moreover, Kerckhoffs et al. (2012) modified the growth law to exclude the “unbounded” growth with a growth multiplier limiting function. Göktepe et al. (2010a) also developed a framework for using the bounded stress- and strain-driven growth laws to study pathological and physiological growth processes in the living heart. One of the debating points in this approach is whether a growth field is compatible. In addition, since soft tissues are essentially fibre-reinforced materials, growth theories that describe changes in the fibre structure have also been put forward, either by directly tracking the micro-fibre structure remodelling (Driessen et al. 2003) or indirectly by including the density growth where the volume of the structure remains constant but its density varies (Eriksson et al. 2014). A few studies considered the evolution of the fibre distribution as well during G&R (Kroon et al. 2007; Rouillard and Holmes 2012; Zhuan et al. 2019).

Of the two categories, the volumetric growth theory has been widely used due to its simplicity. However, a long-standing issue of the volumetric growth theory is that most existing volumetric growth studies assumed that growth occurs in the natural (reference) configuration (Budday et al. 2014; Göktepe et al. 2010a). Under this assumption, the volumetric growth is modelled by introducing a so-called growth tensor defined from the reference configuration (Rodriguez et al. 1994; Hsu 1968; Kerckhoffs et al. 2012; Kerckhoffs 2012; Klepach et al. 2012), so that the overall deformation tensor ${\mathbf{A}}$ can be decomposed as

1 $$\begin{aligned} {\mathbf{A}} ={\mathbf{F} _e} {\mathbf{F} _g}, \end{aligned}$$

where ${\mathbf{F} _g}$ is the pure growth tensor, and ${\mathbf{F} _e}$ is the pure elastic deformation tensor. For fibre-reinforced soft tissue modelling, if the fibre structure in the reference configuration is represented by the fibre, sheet, and normal directions ${\mathbf{f}_0}, {\mathbf{s}_0}, {\mathbf{n} _0}$, then a commonly used growth tensor can be expressed as (Kerckhoffs et al. 2012)

2 $$\begin{aligned} {\mathbf{F}_g} = {\vartheta }^f {\mathbf{f }_0} \otimes {\mathbf{f }_0}+ {\vartheta }^s {\mathbf{s }_0} \otimes {\mathbf{s }_0}+ {\vartheta }^n {\mathbf{n }_0} \otimes {\mathbf{n }_0}, \end{aligned}$$

where ${\vartheta }^j (\mathbf{X},t)$, $(j=f, s, n)$, are called the growth multipliers. Note that growth is assumed to be along the principal cylindrical directions, and are functions of the position vector ${\mathbf{X}}$ of the reference configuration and time *t*. These are smaller or greater than one when the elastic body shrinks or grows with respect to the reference configuration.

If growth is compatible, then no residual stress is generated. This process is discussed in sect. 2.1.1. The evolution of the growth (multipliers) can be either considered as stress-driven (Taber 1998; Lubarda and Hoger 2002; Taber and Chabert 2002; Hariton et al. 2007), strain-driven (Kerckhoffs et al. 2012; Driessen et al. 2004), or combined stress- and strain- driven driven (Taber and Eggers 1996). One important aspect of G&R theories is their thermodynamics consistence, which has been discussed in details by Epstein and Maugin (2000). For growth from the reference configuration, it appears that the Mandel stress plays an important role in thermodynamically consistent formulations of inelasticity. It is for this reason that Mandel stress is often used in stress-driven growth laws.

Amar and Goriely (2005) and Goriely and Amar (2007) moved a step further and studied growth from the current configuration without releasing the residual stress, albeit for simplified cases, such as symmetrical spheres (Goriely and Amar 2007). However, living organs cannot always be so simplified. More recently, Genet et al. (2015) introduced a growth involution law in the updated stress-free configuration. However, they still used the growth tensor defined in the fixed reference configuration, and the strain energy function remained the same during growth. Therefore, it is unclear if their approach is thermodynamically consistent.

In reality, soft tissue G&R is a continuous process, so growth must occur in the current (loaded and residually stressed) configuration. In other words, given the geometrical constraints, pure growth generally induces material incompatibility (Skalak et al. 1996). However, a theoretical framework for an incompatible growth in the current configuration is missing, although some efforts have been made in this direction by studying growth evolution from an updated reference configuration after each incremental growth. For example, an inhomogeneous volumetric growth was studied using a three-dimensional simulation of the heart (Kroon et al. 2009). However, in these studies, it was assumed that residual stress is released after each incremental growth, and that further growth always starts from the updated, yet stress-free configurations. The reason for people to adopt this assumption is that the updated natural configurations can be determined directly from the cumulative incremental growth (or growth history) which leads to a similar computational method to the growth models from the reference configuration (Budday et al. 2014; Göktepe et al. 2010a). However, the cost of this convenience is to force growth to be compatible at all times, which is a major model limitation. Amar and Goriely (2005) and Goriely and Amar (2007) moved a step further and studied growth from the current configuration, albeit for simplified cases, such as symmetrical spheres.

In particular, Goriely and Amar (2007) employed an incremental theory to propose a continuous description of the sequential growth from the current configuration. Although they started with general problems, progress was only made when the growth tensor and the deformation gradient tensor are both assumed diagonal.

More recently, Genet et al. (2015) introduced a growth evolution law in an updated stress-free configuration. However, they still used the growth tensor defined in the fixed reference configuration, and the strain energy function remained the same during the growth. Therefore, it is unclear if their approach is thermodynamically consistent.

In this paper, we propose a new framework of volumetric growth evaluated in the current configuration, which is thermodynamically consistent, and without releasing the residual stress or imposing geometrical and deformational restrictions. In addition, we shall show that our theory can recover the results of Goriely and Amar (2007) under the same simplifications they used. We also establish the relationships between the strain energy functions defined in different configurations, i.e. in the natural, current (stressed), and the evolving, grown, but stress-free configurations. This is different to previous work done by groups, e.g. (Ogden and Saccomandi 2007; Holzapfel and Ogden 2010; Gower et al. 2017; Agosti et al. 2018), who derived the constitutive laws from current stressed configurations for soft tissue mechanics, but not applied to G&R processes.

We illustrate our idea using both a left ventricle (LV) and a cylinder model, which admit an inhomogeneous growth in a residually stressed current configuration. We also show that the total cumulative growth from the reference configuration, the expression of which is prescribed in previous volumetric growth theories, can be derived based on this new framework. This cumulative growth is not affected by elastic stretches but is dependent on elastic rotations. In other words, the cumulative growth tensor is a function of loading history, and hence, cannot be postulated *a priori*. The strain energy function with respect to the updated reference configurations also changes with growth. In this new approach, the stress-driven growth law is taken to be a function of the Cauchy stress tensor (also known as the true stress). Finally, we compare the residual stress of the LV model using growth laws defined in the reference and current configurations, respectively, with published experimental measurements (Costa et al. 1997; Omens et al. 1993), and show that our approach leads to qualitative agreements with the measurements.

## Volumetric growth theories

We take the accepted hypothesis that pure growth does not induce any elastic deformation [H1]. In other words, pure growth and elastic stretching of a body are two independent events. We also assume that the strain energy of a body at any time is a function of the total deformation gradient, which can be a combination of elastic and residual-stress induced deformations [H2] (Ogden 1997).

### Growth from the reference (natural) configuration

We first introduce the concept and notations of existing volumetric growth theories based on the reference (natural) configuration.

#### Compatible growth

**Fig. 1:**
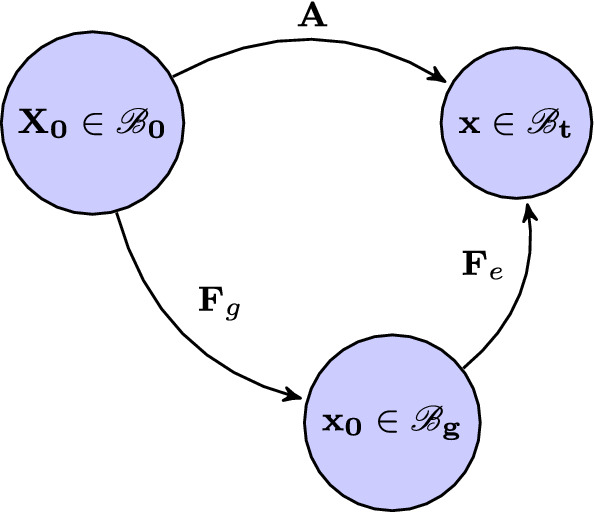
Compatible growth from the reference configuration, ${{\mathscr {B}}_0}$. A pure and compatible growth ${\mathbf{F}_{g}}$ takes the elastic body from ${\mathscr {B}}_0$ into ${\mathscr {B}}_g$, and is then deformed via the loading-induced elastic deformation ${\mathbf{F}_e}$ into the current configuration ${\mathscr {B}}_t$. The total deformation gradient from ${\mathscr {B}}_0$ to ${\mathscr {B}}_t$ is denoted as $\mathbf{A}$

Let ${\mathbf{X}}$ and ${\mathbf{x}}$ be the position vectors of a material point in the reference and current configurations, ${{\mathscr {B}}_0}$ and ${{\mathscr{B}}_t}$, respectively. The idea of growth from the reference configuration is illustrated in Fig. 1, where a pure (compatible) growth ${\mathbf{F} _{g}}$ takes an elastic body from ${{\mathscr {B}}_0}$ to an (imaginary) grown and stress-free configuration ${{\mathscr {B}}_g}$ as ${\mathbf{F} _g=\frac{\partial {\mathbf{x}_0}}{\partial {\mathbf{X}_0}}}$, assuming that the map ${\mathbf{x}_0}{(\mathbf{X}_0)}$ exists and is differentiable. Then, after loading the body deforms into the current configuration ${{\mathscr {B}}_t}$ with elastic deformation gradient ${\mathbf{F} _e}=\frac{\partial \mathbf{x}}{\partial {\mathbf{x}_0}}$.

The overall deformation gradient is

3 $$\begin{aligned} {\mathbf{A}} ={\mathbf{F}}_e{\mathbf{F}_g}. \end{aligned}$$

Following (Ogden 2003), for incompressible materials that undergo volumetric growth, we must have the connection

4 $$\begin{aligned} J^{-1}_{F_e} W_g({\mathbf{F} _e}) =J^{-1}_A[ W_{0}({\mathbf{A}} )-W_{0}( {\mathbf{F} _{g}})], \end{aligned}$$

where $W_0$ is the free energy per unit volume in ${\mathscr {B}}_0$, $W_g$ is the elastic strain energy function per unit volume in ${\mathscr {B}}_g$, $J_A=\det {\mathbf{A}}$, and $J_{F_e}=\det {\mathbf{F}} _{e}$. Since $W_{0}( {\mathbf{F}} _{g}) \equiv 0$ due to the hypothesis [H1], we simply have

5 $$\begin{aligned} W_g({\mathbf{F} _e})=J^{-1}_g W_{0}({\mathbf{A}}), \end{aligned}$$

where we have used $J_A=J_g=\det {\mathbf{F}_{g}}$, and $J_{F_e}=1$.

The Cauchy stress in ${\mathscr {B}}_t$ can be determined either through the total deformation $\mathbf{A}$ from ${\mathscr {B}}_0$, or through the elastic deformation $\mathbf{F} _e$ from ${\mathscr {B}}_g$,

6 $$\begin{aligned} \varvec{\sigma }= J_g^{-1} \mathbf{A} \frac{\partial W_{0}( \mathbf{A} )}{\partial \mathbf{A} } = \mathbf{F} _e \frac{\partial W_g(\mathbf{F} _e)}{\partial \mathbf{F} _e}. \end{aligned}$$

Equation (6) can be proved by making use of (5) and (3), i.e.

7 $$\begin{aligned} \varvec{\sigma }= J_g^{-1} \mathbf{A} \frac{\partial W_{0} (\mathbf{A})}{\partial \mathbf{A} } = \mathbf{F} _e\mathbf{F} _g (\frac{\partial W_g(\mathbf{F} _e)}{\partial \mathbf{F} _e} \frac{\partial \mathbf{F} _e}{\partial \mathbf{A} }) = \mathbf{F} _e \frac{\partial W_g(\mathbf{F} _e)}{\partial \mathbf{F} _e}. \end{aligned}$$

#### Incompatible growth

**Fig. 2:**
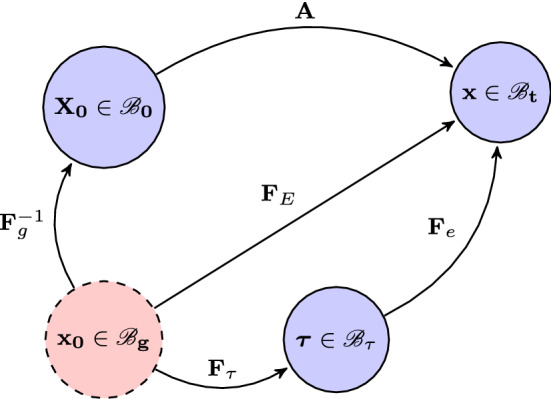
A pure growth $\mathbf{F}_{g}$ from ${\mathscr {B}}_0$ takes the body to an incompatible and stress-free state in configuration ${\mathscr {B}}_g$. The deformation $\mathbf{F}_{\tau }$ assembles the body into a compatible, but residually stressed stated in configuration ${\mathscr {B}}_\tau$. Loading induced deformation $\mathbf{F}_{e}$ then maps it onto the current configuration ${\mathscr {B}}_t$. The process can be equally viewed as $\mathbf{F}^{-1}_g$ from ${\mathscr {B}}_g$ to ${\mathscr {B}}_0$ followed by $\mathbf{A}$ from ${\mathscr {B}}_0$ to ${\mathscr {B}}_t$, or from ${\mathscr {B}}_g$ to ${\mathscr {B}}_t$ directly via the elastic deformation $\mathbf{F}_{E}$

We use the pure growth to describe the process so that if the growth tensor used to designate the growth which would be locally observed at a point of the tissue with the small region around it could be grown in isolation, then this leads to an incompatible strain field, and residual stress is required to keep the tissue intact (Skalak et al. 1996). This process is shown in Fig. 2, where the grown and stress-free configuration ${\mathscr {B}}_g$ is made compatible via an elastic deformation $\mathbf{F} _\tau$ into ${\mathscr {B}}_\tau$ with residually stress, before the loading-induced $\mathbf{F} _e$ maps ${\mathscr {B}}_\tau$ into ${\mathscr {B}}_t$. As shown in Fig. 2 and [H2], this process can also be viewed as a growth-induced residual deformation $\mathbf{F}_\tau$ from ${\mathscr {B}}_g$ to ${\mathscr {B}}_\tau$ followed by $\mathbf{F}_{e}$.

We now focus on incompressible materials and choose ${\mathscr {B}}_g$ as the new (evolving) reference configuration, with a strain energy function $W_g$. The total elastic deformation gradient from ${\mathscr {B}}_g$ to ${\mathscr {B}}_t$ is

8 $$\begin{aligned} \mathbf{F} _E=\mathbf{A} {} \mathbf{F} ^{-1}_g =\mathbf{F} _e \mathbf{F} _\tau . \end{aligned}$$

As before, the Cauchy stress in ${\mathscr {B}}_t$ can be determined either from the reference configuration ${\mathscr {B}}_g$,

9 $$\begin{aligned} \varvec{\sigma }= \mathbf{F} _E \frac{\partial W_{g}(\mathbf{F} _E)}{\partial \mathbf{F} _E}-p\mathbf{I}, \end{aligned}$$

where $W_{g}$ is the elastic strain energy function related to the configuration ${\mathscr {B}}_g$.

or from $${\mathscr {B}}_\tau $$,

10 $$\begin{aligned} \varvec{\sigma }= \mathbf{F} _e \frac{\partial W_\tau ( \mathbf{F} _e, {\varvec{\tau }})}{\partial \mathbf{F} _e}-p^\tau \mathbf{I}. \end{aligned}$$

where the strain energy function $W_\tau$ from ${\mathscr {B}}_\tau$ is a function of both $\mathbf{F}_{e}$ and residual stress $\varvec{\tau }$, and *p*, $p^\tau$ are the Lagrange multipliers to ensure incompressibility of the material. The identity ($p=p^\tau$) has been proved by (Ogden 2003). Henceforth, we only use *p*.

The equivalence of (9) and (10) can be similarly proved using the connection,

11 $$\begin{aligned} W_{\tau }(\mathbf{F} _{e}, \varvec{\tau }) = W_g (\mathbf{F} _E)- W_g(\mathbf{F} _{\tau }). \end{aligned}$$

Substituting (5) and (11) into (10), and making use of [H1], so that $\partial \mathbf{F} _\tau /\partial \mathbf{F} _e = \mathbf{0}$, we obtain (9).

When evaluated in ${\mathscr {B}}_{\tau }$ with $\mathbf{F} _e=\mathbf{I}$ and $\mathbf{F} _E=\mathbf{F _\tau }$, the Cauchy stress $\varvec{\sigma }$ simply becomes the residual stress $\varvec{\tau }$, i.e.

12 $$\begin{aligned} \varvec{\tau }= \mathbf{F} _\tau \frac{\partial W_{g}( \mathbf{F} _\tau ) }{\partial \mathbf{F} _\tau }-p\mathbf{I} = \frac{\partial W_{\tau }( \mathbf{I} , \varvec{\tau })}{\partial \mathbf{F} _e}-p\mathbf{I}. \end{aligned}$$

The residual stress and the Cauchy stress must obey their corresponding equilibrium equations,

13 $$\begin{aligned} \text {div} {\varvec{\tau }}=\mathbf{0} \quad \text {or} \quad \text {div} {\varvec{\sigma }}=\mathbf{0}. \end{aligned}$$

The boundary conditions are zero-loading for ${\varvec{\tau }}$ and loading for ${\varvec{\sigma }}$, respectively. This enables us to solve for $\mathbf{F} _{\tau }$ and $\mathbf{F} _{e}$, and hence, $\varvec{\tau }$ and $\varvec{\sigma }$.

### Growth evaluated in the current (stressed) configuration

**Fig. 3:**
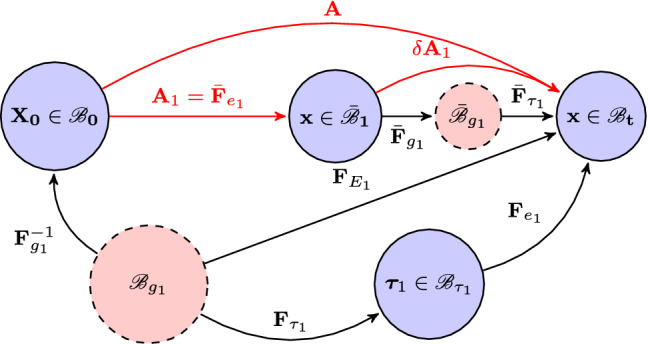
Growth from a stressed configuration $\bar{{\mathscr {B}}_1}$ following an elastic deformation $\bar{\mathbf{F}}_{e_1}$. Then, the body undergoes a small incremental deformation $\delta \mathbf{A} _1=\bar{\mathbf{F}}_{\tau _1}\bar{\mathbf{F}}_{g_1}$ from $\bar{{\mathscr {B}}_1}$ to ${\mathscr {B}}_{t}$. The process can be equally viewed as $\mathbf{F}^{-1}_{g_1}$ from ${\mathscr {B}}_{g_1}$ to ${\mathscr {B}}_0$ followed by $\mathbf{A}$ from ${\mathscr {B}}_0$ to ${\mathscr {B}}_t$, or from ${\mathscr {B}}_{g_1}$ to ${\mathscr {B}}_t$ directly via the elastic deformation $\mathbf{F}_{E_1}$

We now propose a new theory that enables growth law to be evaluated in the current (stressed) configuration. We denote such a configuration as $\bar{{\mathscr {B}}}_1$. As shown in Fig. 3, $\bar{{\mathscr {B}}}_1$ is reached after the body is deformed from the reference configuration ${\mathscr {B}}_0$.

Let $\mathbf{A} _1=\bar{\mathbf{F }}_{e_1}$ indicate this elastic deformation. Since the line elements of material transform according to $d \mathbf{x}=\mathbf{A}_1 d \mathbf{X_0}$, we have the incremental connection,

14 $$\begin{aligned} \delta (d\mathbf{x} )=\delta \mathbf{A}_1(d\mathbf{X} _0). \end{aligned}$$

If the stressed configuration $\bar{{\mathscr {B}}}_1$ is now chosen as the reference configuration then the right-hand side of (14) becomes $\delta \mathbf{A_0}(d\mathbf{x} )$, where $\delta \mathbf{A_0}$ is the value of $\delta \mathbf{A}_1$ in this configuration. Here, $\mathbf{A}_0$ is the unit tensor before the initial configuration ${\mathscr {B}}_0$: $\mathbf{A}_0=I$. Since $d\mathbf{x}$ and hence $\delta (d\mathbf{x} )$ is independent of the reference configuration, we obtain the connection (Ogden 1997)

15 $$\begin{aligned} \delta \mathbf{A} _0 =\delta \mathbf{A} _1 \mathbf{A} _1. \end{aligned}$$

Now consider an incremental deformation caused by the pure growth $\bar{\mathbf{F }}_{g_1}$ and $\bar{\mathbf{F}}_{\tau _1}$, which take the body into the incompatible configuration $\bar{{\mathscr {B}}}_{g_1}$ first and then assemble it into a compatible configuration $\bar{{\mathscr {B}}}_{\tau _1}$. If the growth displacement $\delta \mathbf{x}$ is “small” for each $\mathbf{X}$ in ${\mathscr {B}}_0$ so that terms of order $\ge |\delta \mathbf{x} |^2$ are negligible in comparison with those of order $|\delta \mathbf{x} |$ then we refer to $\delta \mathbf{A} _1=\bar{\mathbf{F}}_{\tau _1}\bar{\mathbf{F}}_{g_1}$ as an incremental (growth) deformation from $\bar{{\mathscr {B}}}_1$ to ${\mathscr {B}}_t$.

This approach is the classic growth incremental procedure (i.e. in Amar and Goriely (2005); Göktepe et al. (2010a)). Goriely and Amar (2007) also employed the similar approach but included the second-order deformation increments.

Since $\delta \mathbf{A} _0 =\mathbf{A}$, $\delta \mathbf{A} _1=\bar{\mathbf{F}}_{\tau _1}\bar{\mathbf{F}}_{g_1}$, $\mathbf{A} _1=\bar{\mathbf{F}}_{e1}$, (15) can also be written as

16 $$\begin{aligned} \mathbf{A} =\bar{\mathbf{F}}_{\tau _1}\bar{\mathbf{F}}_{g_1} \bar{\mathbf{F}}_{e_1}. \end{aligned}$$

As in Sect. 2.1.2, the total elastic deformation gradient $\mathbf{F} _{E_1}$ can be equally achieved either along the top path ${\mathscr {B}}_{g_1}$–${\mathscr {B}}_{0}$–${\mathscr {B}}_t$, or the lower path ${\mathscr {B}}_{g_1}$–${\mathscr {B}}_{\tau _1}$–${\mathscr {B}}_t$. The physical interpretation of the lower path ${\mathscr {B}}_{g_1}$–${\mathscr {B}}_{\tau _1}$–${\mathscr {B}}_t$ is that a pure growth $\mathbf{F}_{g_1}$, which can be derived from $\bar{\mathbf{F}}_{g_1}$, maps the reference configuration ${\mathscr {B}}_0$ into the grown and incompatible (stress free) configuration ${\mathscr {B}}_{g_1}$. ${\mathscr {B}}_{g_1}$ is made compatible by $\mathbf{F}_{\tau _1}$ and becomes ${\mathscr {B}}_{\tau _1}$ with the residual stress $\mathbf {\tau }_1$. From ${\mathscr {B}}_{\tau _1}$ the body is deformed to ${\mathscr {B}}_{t}$ by the elastic deformation $\mathbf{F}_{e_1}$. The process of the top path can be viewed as $\mathbf{F}^{-1}_{g_1}$ from ${\mathscr {B}}_{g_1}$ to ${\mathscr {B}}_0$ followed by $\mathbf{A}$ from ${\mathscr {B}}_0$ to ${\mathscr {B}}_t$. So again, we have

17 $$\begin{aligned} \mathbf{F} _{E_1}=\mathbf{A} {} \mathbf{F} ^{-1}_{g_1} =\mathbf{F} _{e_1} \mathbf{F} _{\tau _1}, \end{aligned}$$

from which we obtain

18 $$\begin{aligned} \mathbf{A} =\mathbf{F} _{e_1} \mathbf{F} _{\tau _1}{} \mathbf{F} _{g_1}. \end{aligned}$$

We remark that for growth from the current configuration, it is not easy to follow the top path in Fig. 3, since we do not introduce the strain energy function with respect to $\bar{{\mathscr {B}}}_{g_1}$ or remove the external loading. On the other hand, if we follow the lower two paths, we have the situation already discussed in Sect. 2.1.2. In other words, we always compute $\mathbf{A}$ from (18), and not from (16). However, to use (18), we must first establish the connection between $\bar{\mathbf{F }}_{g_1}$ and $\mathbf{F} _{g_1}$.

Since pure growth is independent of the elastic stretch (H1), the only difference between $\bar{\mathbf{F}}_{g_1}$ and $\mathbf{F} _{g_1}$ is due to the rotation of $\bar{\mathbf{F}}_{e_1}$, i.e.

19 $$\begin{aligned} \mathbf{F} _{g_1} = (\bar{\mathbf{R }}_{r_1})^\text {T} \bar{\mathbf{F }}_{g_1} \bar{\mathbf{R }}_{r_1}. \end{aligned}$$

where $\bar{\mathbf{R}}_{r_1}= \bar{\mathbf{V}}_{e_1}^{-1}\bar{\mathbf{F}}_{e_1}$. Since $\bar{\mathbf{F}}_{e_1}$ can be easily computed, given $\bar{\mathbf{F }}_{g_1}$, $\mathbf{F }_{g_1}$ is known.

The rest of the analysis simply follows the approach of Sect. 2.1.2. The Cauchy stress in ${\mathscr {B}}_t$ is

20 $$\begin{aligned} \varvec{\sigma }_1= \mathbf{F} _{E_1} \frac{\partial W_{g_1}(\mathbf{F} _{E_1})}{\partial \mathbf{F} _{E_1}}-p\mathbf{I} = \mathbf{F} _{e_1} \frac{\partial W_{\tau _1}( \mathbf{F} _{e_1}, {\varvec{\tau }})}{\partial \mathbf{F} _{e_1}}-p\mathbf{I}. \end{aligned}$$

The strain energy function $W_{\tau _1}$ with respect to ${\mathscr {B}}_{\tau _1}$ satisfies

21 $$\begin{aligned} W_{\tau _1}(\mathbf{F} _{e_1}, \varvec{\tau }_1 ) = W_{g_1} (\mathbf{F} _{E_1})- W_{g_1}(\mathbf{F} _{\tau _1}). \end{aligned}$$

The residual stress $\varvec{\tau }_1$ is simply the Cauchy stress evaluated in ${\mathscr {B}}_{\tau _1}$ when the external loading is removed, i.e. $\mathbf{F} _{e_1}=\mathbf{I}$, $\mathbf{F} _{E_1}=\mathbf{F} _{\tau _1}$. Hence,

22 $$\begin{aligned} \varvec{\tau }_1 = \mathbf{F} _{\tau _1} \frac{\partial W_{g_1}( \mathbf{F} _{\tau _1}) }{\partial \mathbf{F} _{\tau _1}}-p\mathbf{I} = \frac{\partial W_{\tau _1}( \mathbf{I} , \varvec{\tau }_1)}{\partial \mathbf{F} _{e_1}}-p\mathbf{I}. \end{aligned}$$

Again, $\varvec{\tau }_1$ and $\varvec{\sigma }_1$ both obey the corresponding equilibrium equations, and the appropriate boundary conditions. From these, we solve for $\mathbf{F} _{\tau _1}$ and $\mathbf{F} _{e_1}$, and $\varvec{\tau _1}$ and $\varvec{\sigma _1}$.

### Subsequent growth from stressed and grown configuration

**Fig. 4:**
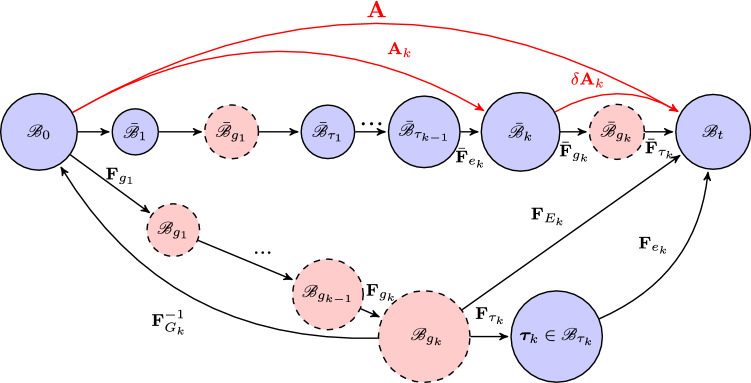
General roadmap after *k* steps of continuous growth and deformation. The deformation gradient from ${{\mathscr {B}}}_{0}$ to ${{\mathscr {B}}}_{t}$ is $\mathbf{A}$, the incremental growth from the loaded configuration $\bar{{\mathscr {B}}}_{k}$ is $\delta \mathbf{A}_k=\bar{\mathbf{F }}_{\tau _k}\bar{\mathbf{ F }}_{g_k}$. The total cumulative pure growth tensor ${{\mathscr {B}}}_{0}$ to ${{\mathscr {B}}}_{g_k}$ is $\mathbf{F}_{G_k}$. ${\mathscr {B}}_{g_k}$ is the grown, stress free but incompatible configuration, and ${\mathscr {B}}_{\tau _k}$ is the compatible and residually stressed configuration

Subsequent growth can occur for *k* steps of growth and deformation in $\bar{{\mathscr {B}}}_{k}$, as shown in Fig. 4. In general, external loading may also change after every step. Now with an new incremental growth $\delta \mathbf{A} _k$ from $\bar{{\mathscr {B}}}_{k}$, the total deformation gradient from ${\mathscr {B}}_0$ to ${\mathscr {B}}_t$ becomes

23 $$\begin{aligned} \mathbf{A} = \delta \mathbf{A} _k \mathbf{A} _{k} = \mathbf{F }_{e_k}\mathbf{F }_{\tau _k}, \end{aligned}$$

where $$\delta \mathbf{A} _{k}=\bar{\mathbf{F }}_{\tau _k} \bar{\mathbf{F }}_{g_k}$$,

24 $$\begin{aligned} \mathbf{A} _{k}= \bar{\mathbf{F }}_{e_k}\bar{\mathbf{F }}_{\tau _{k-1}} \bar{\mathbf{F }}_{g_{k-1}}\bar{\mathbf{F }}_{e_{k-1}} ...\bar{\mathbf{F }}_{\tau _1} \bar{\mathbf{F }}_{g_1} \bar{\mathbf{F }}_{e_1} = (\delta \mathbf{A}_k)^{-1}\mathbf{F }_{e_k}\mathbf{F }_{\tau _k} \end{aligned}$$

is the deformation gradient from ${\mathscr {B}}_0$ to $\bar{{\mathscr {B}}}_k$, and

25 $$\begin{aligned} \mathbf{F} _{E_k}=\mathbf{A} {} \mathbf{F} ^{-1}_{G_k} =\mathbf{F} _{e_k} \mathbf{F} _{\tau _k}. \end{aligned}$$

Here, $\mathbf{F} _{G_k}$ is the total cumulative pure growth from ${\mathscr {B}}_0$ to ${\mathscr {B}}_{g_k}$, computed from the previous cumulative pure growth $\mathbf{F} _{G_{k-1}}= \mathbf{F} _{g_{k-1}}...\mathbf{F} _{g_1}$, i.e.

26 $$\begin{aligned} \mathbf{F} _{G_k} =\mathbf{F} _{g_k}{} \mathbf{F} _{G_{k-1}}. \end{aligned}$$

Again, to follow the path ${\mathscr {B}}_{g_k}$–${\mathscr {B}}_{\tau _k}$–${\mathscr {B}}_t$, we must first find the connection between $\bar{\mathbf{F }}_{g_k}$ and $\mathbf{F} _{g_k}$, so that (26) can be evaluated. The deformation gradient between ${{{\bar{B}}}}_{k}$ and ${\mathscr {B}}_{gk}$ is ${\mathbf{A} _{k}} {\mathbf{F} ^{-1}_{G_{k-1}}}$. Let ${\mathbf{F} _{r_k}}={\mathbf{A} _{k}}{} {\mathbf{F} ^{-1}_{G_{k-1}}}$, then we have the connection

27 $$\begin{aligned} \mathbf{F} _{g_k}= (\mathbf{R} _{r_k})^\text {T} \bar{\mathbf{F }}_{g_k} \mathbf{R} _{r_k}, \end{aligned}$$

where $\mathbf{R} _{r_k}$ is the rotation of $\mathbf{F} _{r_k}$.

The procedure is now the same as before. So we give the general expressions. The Cauchy stress in ${\mathscr {B}}_t$ is

28 $$\begin{aligned} \varvec{\sigma }_k= \mathbf{F} _{E_k} \frac{\partial W_{g_k}(\mathbf{F} _{E_k})}{\partial \mathbf{F} _{E_k}}-p\mathbf{I} = \mathbf{F} _{e_k} \frac{\partial W_{\tau _k}( \mathbf{F} _{e_k}, {\varvec{\tau }})}{\partial \mathbf{F} _{e_k}}-p\mathbf{I}, \end{aligned}$$

where

29 $$\begin{aligned} W_{\tau _k}(\mathbf{F} _{e_k}, \varvec{\tau }_k ) = W_{g_k} (\mathbf{F} _{E_k})- W_{g_k}(\mathbf{F} _{\tau _k}). \end{aligned}$$

Similar to (22), the residual stress $\varvec{\tau _k}$ is

30 $$\begin{aligned} \varvec{\tau }_k = \mathbf{F} _{\tau _k} \frac{\partial W_{g_k}( \mathbf{F} _{\tau _k}) }{\partial \mathbf{F} _{\tau _k}}-p\mathbf{I} = \frac{\partial W_{\tau _k}( \mathbf{I} , \varvec{\tau }_k)}{\partial \mathbf{F} _{e_k}}-p\mathbf{I}. \end{aligned}$$

Again, $\varvec{\tau }_k$ and $\varvec{\sigma }_k$ both obey the corresponding equilibrium equations and the appropriate boundary conditions. From these, we can solve for $\mathbf{F} _{\tau _k}$ and $\mathbf{F} _{e_k}$.

At this point, all we need is an incremental growth law for $\bar{ \mathbf{F}}_{g_k}$.

### Incremental growth law

If we consider continuous growth and deformation, then we can write $\mathbf{F} _{g_k}=\mathbf{F} _{g_t}$, similarly, $\mathbf{F} _{G_k} =\mathbf{F} _{G_t}$. For simplicity, we assume that the growth tensor is diagonal for a fibre-reinforced material. This assumption is widely accepted by the community and seems to hold for the left ventricle (Li et al. 2021). Consequently, all the incremental growth tensors are also diagonal. Note it is not essential to assume a diagonal incremental growth tensor in this framework. A more general growth law including off-diagonal elements can equally work if one has experimental data to support the expression. However, the assumption is used to illustrate the framework more clearly, and to compare our results with those from previous work. Let $\bar{\mathbf{F}}_{g_t}=d{\varvec{\vartheta }}$ be the incremental growth tensor, we write

31 $$\begin{aligned} \bar{\mathbf{F}}_{g_t}/dt={\dot{\vartheta }}^f \mathbf{f }_t \otimes \mathbf{f }_t+ {\dot{\vartheta }}^s \mathbf{s }_t \otimes \mathbf{s }_t+ {\dot{\vartheta }}^n \mathbf{n }_t \otimes \mathbf{n }_t, \end{aligned}$$

where ($\mathbf{f} _t, \mathbf{s} _t,\mathbf{n} _t$) is the fibre structure of the material at time *t*, which is related to the fibre structure at $t=0$ via

32 $$\begin{aligned} {\mathbf {f}}_{t} = \frac{\mathbf {A} {\mathbf {f}}_0}{||\mathbf {A} {\mathbf {f}}_0||}, \ {\mathbf {s}}_{t} = \frac{\mathbf {A} {\mathbf {s}}_0}{||\mathbf {A} {\mathbf {s}}_0||}, \ {\mathbf {n}}_t = \frac{\mathbf {A} {\mathbf {n}}_0}{||\mathbf {A} {\mathbf {n}}_0||}, \end{aligned}$$

and ${\dot{\vartheta }}^f, {\dot{\vartheta }}^s,{\dot{\vartheta }}^n$ in (31) are the rates of change of the growth multipliers in the $\mathbf{f} _t, \mathbf{s} _t,\mathbf{n} _t$ directions, respectively, which can be written as $\dot{\varvec{\vartheta }} (\mathbf{x},t)= \left[ {\dot{\vartheta }}^f, {\dot{\vartheta }}^s,{\dot{\vartheta }}^n \right]$. Note $\varvec{\vartheta }= \text {diag} (\mathbf{F} _{G_t})$, so $\varvec{\vartheta }$ represents the total cumulative growth vector of the elastic body with respect to the reference configuration ${\mathscr {B}}_0$.

We now assume that growth can be either stress or strain driven. Using a similar evolution law as in (Lubarda and Hoger 2002; Göktepe et al. 2010a), we write

33 $$\begin{aligned}&\text {diag} (\bar{\mathbf{F}}_{g_t}/dt) = \dot{\varvec{\vartheta }} = \varvec{l} (\varvec{\vartheta }) \phi (\varvec{\sigma }_t) \quad \text {or} \nonumber \\&\quad \text {diag} (\bar{\mathbf{F}}_{g_t}/dt)= \dot{\varvec{\vartheta }} = \varvec{l} (\varvec{\vartheta }) \phi (\mathbf{e} _t), \end{aligned}$$

where $\varvec{\sigma }_t$ and $\mathbf{e}_t=\frac{1}{2} (\mathbf {I}-\mathbf {A}^{-\text {T}}\mathbf {A}^{-1})$ are the Cauchy stress and Eulerian strain tensors in ${\mathscr {B}}_t$, respectively.

The growth threshold $\phi$ is a scalar function of either the Cauchy stress tensor or the Eulerian strain tensor, depending if growth is stress or strain driven. In other words, growth is activated once the stress or strain during physical activity exceeds the physiological threshold level. If $\phi$ is negative, then there is no growth. $\varvec{l}$=$\left[ l^f, l^s,l^n \right]$ is a limiting or scaling vector function since growth cannot continue infinitely. In this paper, we follow Lubarda and Hoger (2002); Göktepe et al. (2010a) to choose

34 $$\begin{aligned} l^j(\varvec{\vartheta })= \frac{1}{\beta _j} \left[ \frac{\vartheta ^{j\text {max}} -\vartheta ^j}{\vartheta ^{j\text {max}} -1} \right] ^{\gamma _j}, \quad j=f,s,n, \end{aligned}$$

where $\vartheta ^{j\text {max}}$ are the maximum values of the growth multipliers $\vartheta ^{j}$, and $\beta _j$, $\gamma _j$ are growth parameters. The choice of $\phi$ and $l^j(\varvec{\vartheta })$ in (33) will ensure the growth rate changes smoothly until the growth multiplier $\vartheta ^{j}$ has reached its maximum value $\vartheta ^{j\text {max}}$. The nonlinearity and the speed of the growth are governed by $\beta _j$ and $\gamma _j$, respectively.

### The strain energy function

We choose the grown and stress-free configuration ${\mathscr {B}}_{g_t}$ as the reference configuration which evolves with time. We also adopt a modified Holzapfel–Ogden (HO) model for the fibre-reinforced material (Holzapfel and Ogden 2009) in ${\mathscr {B}}_{g_t}$,

35 $$\begin{aligned}W_{g_t} (\mathbf{F}_{E_t}) = & \frac{a}{2b}\left\{ \text {exp}[b({I}_1-3)] -1 \right\} \nonumber \\\quad  + &\ \frac{a_{\text {f}}}{2b_{\text {f}}}\left\{ \exp [ b_{\text {f}}(I_4(\varTheta _g, \varXi _g)-1)^2] -1 \right\} , \end{aligned}$$

where ${I}_1$, $I_4$ are invariants of $\mathbf {C}_{E_t} = \mathbf {F}_{E_t}^\text {T}\mathbf {F}_{E_t}$, the right Cauchy–Green tensor with respect to ${\mathscr {B}}_{g_t}$, $\mathbf {F}_{E_t}$ is the total elastic deformation from ${\mathscr {B}}_{g_t}$ to ${\mathscr {B}}_{t}$,

$$I_1=\mathbf {C}_{E_t}:\mathbf{I} $$ and $${I}_4=\mathbf{f} _{g_t}(\varTheta _g) \cdot \mathbf{C} _{E_t}{} \mathbf{f} _{g_t}(\varTheta _{g_t})$$.

$\varTheta _{g_t}$ and $\varXi _{g_t}$ are angles between $\mathbf{f} _{g_t}$ and $\mathbf{c} _0$, and $\mathbf{f} _{g_t}$ and $\mathbf{n} _0$ in the $\mathbf{c} _0-\mathbf{l} _0$ plane, respectively, and

the fibre direction in ${\mathscr {B}}_{g_t}$ changes according to

36 $$\begin{aligned} \mathbf{f }_{g_t} = \frac{\mathbf {F}^{-1}_{G_t} \mathbf{f }_0}{||\mathbf {F}^{-1}_{G_t} \mathbf{f }_0||}, \ \mathbf{s }_{g_t} = \frac{\mathbf {F}^{-1}_{G_t} \mathbf{s }_0}{||\mathbf {F}^{-1}_{G_t} \mathbf{s }_0||}, \ \mathbf{n }_{g_t} = \frac{\mathbf {F}^{-1}_{G_t} \mathbf{n }_0}{||\mathbf {F}^{-1}_{G_t} \mathbf{n }_0||}. \end{aligned}$$

And *a*, *b* $a_{\text {1}}$, $b_{\text {1}}$ are material parameters.

At $t=0$ there is no growth, ${\mathscr {B}}_{g_0}$ overlaps with ${\mathscr {B}}_{0}$, so $W_{g_0}= W_{0}$.

## Numerical methods

### Finite element model and numerical algorithm

**Fig. 5:**
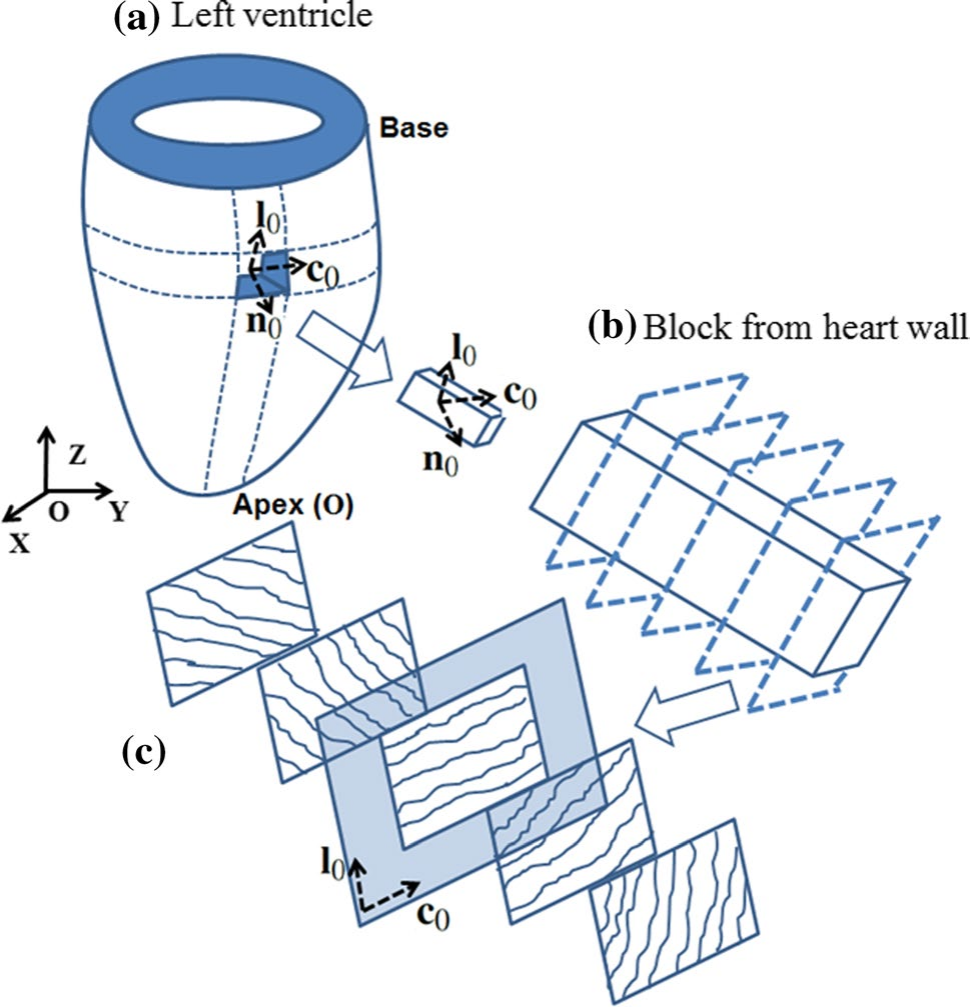
**a** The LV geometry with 28mm long axis, an internal radius of 5mm and external radius of 10mm at the base, and a block cut from the LV wall. The ratio between the LV wall thickness and the internal radius is chosen to be one following Omens and Fung (1990). The basis vectors at the reference configuration are ($\mathbf{c} _0$, $\mathbf{l} _0$, $\mathbf{n} _0$) for local coordinates, where $\mathbf {c}_0$, $\mathbf {l}_0$, and $\mathbf {n}_0$ are the local circumferential, longitudinal and transmural unit vectors. The basis vectors at the reference configuration are ($\mathbf{X}$, $\mathbf{Y}$, $\mathbf{Z}$) for global Cartesian coordinates, with origin **O** at the LV apex. **b** The fibre structure through the thickness of the LV wall. **c** Five longitudinal–circumferential sections through the wall thickness. Collagen fibres lie in the $\mathbf{c} _0$–$\mathbf{l} _0$ plane

The LV diastolic dynamics with growth from loaded configuration is simulated using the finite element package FEAP 8.3, with 2100 8-node hexahedral elements and 2375 nodes (Fig. 5). In addition to the equilibrium equations and boundary conditions, where we fix all the nodes on the base, we also need to solve the evolution equation (33). Since the continuous growth occurs at a time scale much slower than the time for the system to reach the equilibrium, the equations are quasi-time-dependent.

At the current time *t*, after *k* steps of growth.

The growth evolution equation (33) predicts the incremental growth

37 $$\begin{aligned} \text {diag} (\bar{\mathbf{F}}_{g_t}) = \varvec{l} (\varvec{\vartheta }_k ) \phi (\varvec{\sigma }_k)\varDelta t \quad \text {or} \quad \text {diag} (\bar{\mathbf{F}}_{g_t}) = \varvec{l} (\varvec{\vartheta }_k ) \phi (\mathbf{e} _k)\varDelta t, \end{aligned}$$

where $$\varDelta t =t-t_{k}$$.

At every time step, we use a mixed variational approach in FEAP to solve the mechanical behaviour of the nearly incompressible soft tissue that is free from locking. For each element, FEAP uses the Hu–Washizu variational principle

38 $$\begin{aligned} \begin{aligned} \varPi (\mathbf{u} , \hat{\varvec{\sigma }}, \hat{\varvec{\epsilon }}) =&\frac{1}{2} \int _\varOmega \hat{\varvec{\epsilon }}^T {\mathbb {C}}^{(2)}\hat{\varvec{\epsilon }}\ \text {d} \varOmega + \int _\varOmega \hat{\varvec{\sigma }}^T (\nabla ^{(s)}{} \mathbf{u} - \hat{\varvec{\epsilon }}) \text {d} \varOmega \\&- \int _{\partial \varOmega ^\mathbf{u }} \mathbf{t} ^T (\mathbf{u} -\mathbf{u} ^b ) \text {d} \varOmega - \int _{\partial \varOmega ^{\varvec{\sigma }}} \mathbf{u} ^T \mathbf{t} ^b \text {d} \varOmega \end{aligned} \end{aligned}$$

where $\hat{\varvec{\sigma }}$, $\hat{\varvec{\epsilon }}$ are the element ordered vectors of stress and strain tensors, **u** is the displacement vector, ${\mathbb {C}}^{(2)}$ is the ordered matrix of tangent moduli tensor $\mathbf{c} ^{(4)}$ (see (Taylor 2014) for details of the definition of an ordered matrix), **t** is the traction, $\nabla ^{(s)}{} \mathbf{u}$ is the symmetric part of $\nabla \mathbf{u}$, $\mathbf{t} ^b$ is the traction on the force boundary $\partial \varOmega ^{\varvec{\sigma }}$, $\mathbf{u} ^b$ is the displacement vector on the displacement boundary $\partial \varOmega ^\mathbf{u }$. The computational algorithm for the volumetric growth is given below.

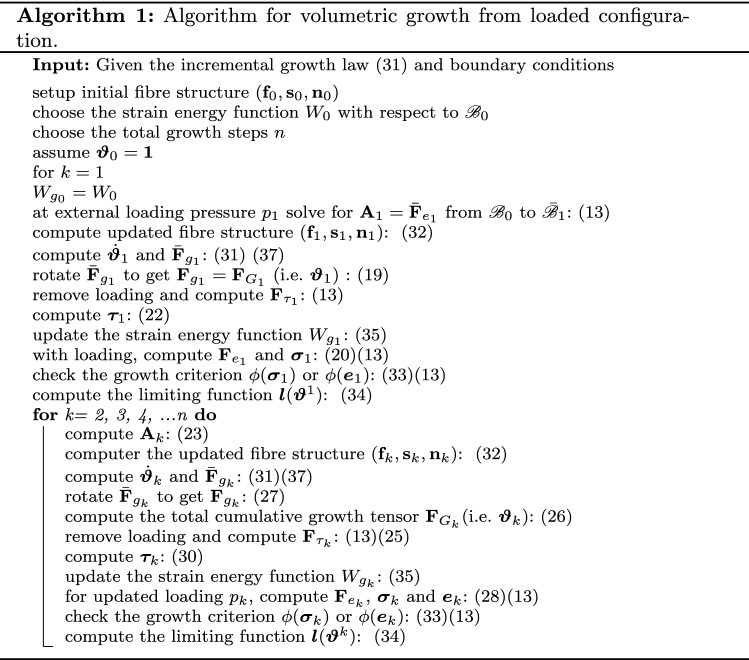



## Test examples

We now use the following examples to illustrate our approach. Henceforth, we use *Growth*-${\mathscr {B}}_0$ to refer to the volumetric growth from the reference configuration, and *Growth*-${\mathscr {B}}_{t}$ to indicate the growth from the current configuration.

### Modelling G&R in myocardium

#### Geometry of the LV model

Myocardium is considered to be a fibre-reinforced material composed of collagen fibres and myocytes.

A local coordinate system with the circumferential, longitudinal, and transmural basis vectors ($\mathbf {c}_0$, $\mathbf {l}_0$, $\mathbf {n}_0$), is introduced to describe the layered fibre structure within the ventricular wall, as shown in Fig. 5. An idealized half ellipsoid geometry is used for a rat LV. Global Cartesian coordinates (*X*, *Y*, *Z*) are used to describe material points in the undeformed reference configuration, with the corresponding basis vectors denoted $\{\mathbf {E}_X, \mathbf {E}_Y, \mathbf {E}_Z \}$. Note that

39 $$\begin{aligned} \mathbf {c}_0= \mathbf {E}_Z \times \mathbf {n}_0, \quad \mathbf {l}_0= \mathbf {n}_0 \times \mathbf {c}_0. \end{aligned}$$

Let the myofibre architecture be described by a “fibre–sheet–normal” system ($\mathbf {f}_0$, $\mathbf {s}_0$, $\mathbf {n}_0$) in the reference configuration, ${\mathscr {B}}_{0}$ (Wang et al. 2013). Here, we assume that the fibre direction $\mathbf {f}_0$ always lies in the $\mathbf{c} _0$–$\mathbf{l} _0$ plane, the sheet direction is transmural, and the sheet–normal $\mathbf {n}_0=\mathbf {f}_0\times \mathbf {s}_0$.

We consider two cases of growth in the myocardium, one is the isotropic growth of athletic’s heart, and the other is the growth following a pathological cardiac dilation. For motivation and physiology, please refer to Göktepe et al. (2010a), who first considered these cases. In both cases, to simulate the LV growth under loading, we use the strain energy function defined in (35), with parameters chosen to be $a=2.28$ kPa, $b=1.8$. We also impose a constant internal pressure of 12 mmHg from $t=0$ throughout the growth.

#### LV case 1: athletic heart and isotropic growth

Since this is isotropic growth, we have ${\dot{\vartheta }}^n={\dot{\vartheta }}^s={\dot{\vartheta }}^f={\dot{\vartheta }}$ such that (31) becomes

40 $$\begin{aligned} \bar{\mathbf{F}}_{g_t}/dt={{\dot{\vartheta }}} \mathbf{I} . \end{aligned}$$

We note in this special case, the fibre structure ($\mathbf{f} _t, \mathbf{n} _t, \mathbf{n} _t$) in the updated stress-free configuration ${\mathscr {B}}_k$ is the same as the initial fibre structure ($\mathbf{f} _0, \mathbf{n} _0, \mathbf{n} _0$).

For *Growth*-${\mathscr {B}}_{0}$, we follow Göktepe et al. (2010a) by assuming the growth is stress driven, using the growth tensor defined in (2), together with the evolution law

$$\begin{aligned} \mathbf{F }_{g}={{\vartheta }} \mathbf{I} , \ \ \ {\dot{\vartheta }} = l (\vartheta ) \phi (\mathbf{M} ), \end{aligned}$$

where

41 $$\begin{aligned} l(\vartheta )= \frac{1}{\beta } \left[ \frac{\vartheta ^{max}-\vartheta }{\vartheta ^{max}-1} \right] ^\gamma , \quad \text {and} \quad \phi = \text {tr}(\mathbf{M} ) - \text {M}^{\text {crit}}. \end{aligned}$$

Here $\mathbf{M}$ is the Mandel stress tensor, $\mathbf{M} =\mathbf{C} {} \mathbf{P}$, $\mathbf{C} =\mathbf{A} ^{\text {T}}{} \mathbf{A}$, $\mathbf{P}$ is the second Piola–Kirchhoff stress tensor, and $\text {M}^\text {crit}$ is a critical stress value. The parameters are chosen as

$$\begin{aligned} \text {M}^\text {crit}=0.0012~{\text{ M }Pa}, \quad \vartheta ^{max} = 1.75, \quad \gamma = 2, \quad \text {and} \quad \beta =1. \end{aligned}$$

For *Growth*-${\mathscr {B}}_{t}$, we have the growth tensor (40) and its evolution law defined in ${\mathscr {B}}_{t}$, and (33)$_1$ and (34), with

$$\begin{aligned} \phi (\varvec{\sigma })= \text {tr}(\varvec{\sigma }) - \sigma ^{\text {crit}}. \end{aligned}$$

and similarly choose

$$\begin{aligned} \sigma ^\text {crit}=0.0012{\text{ M }Pa}, \end{aligned}$$

with all other parameters being the same as in *Growth*-${\mathscr {B}}_{0}$.

**Fig. 6:**
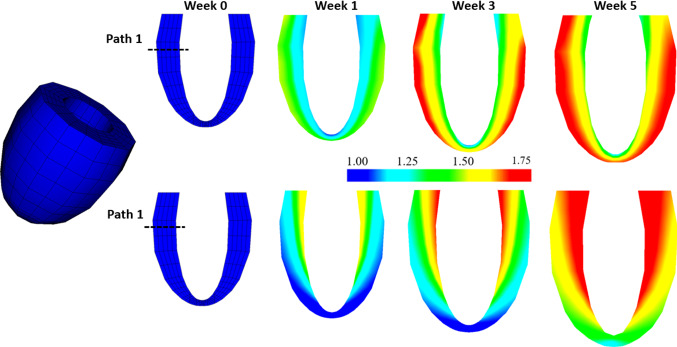
Contours of the growth factor $\vartheta$ in the Case 1 after 0, 1, 3, and 5 weeks growth. Above: Growth-${\mathscr {B}}_0$. Blow: Growth-${\mathscr {B}}_t$. Path 1 at the upper middle section of the LV is defined to compare the transmural variation of results, see text for details

Figure 6 shows that both models display ventricular dilation and wall thickening.

However, the growth patterns are clearly different. In *Growth*-${\mathscr {B}}_{0}$, most of the growth is focused on the apex area, which is opposite to that of the *Growth*-${\mathscr {B}}_{t}$ model. Away from the apex area, the growth is also very different. To make more detailed comparisons, we define a transmural path 1 (see Fig. 6) at the same location for both models.

**Fig. 7:**
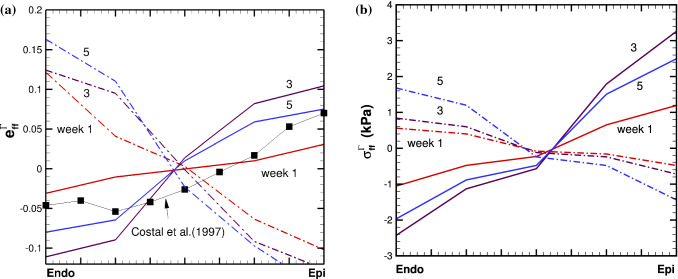
Transmural distribution of the Eulerian residual strain $e_{ff}$ (**a**), and stress ${\tau }_{ff}$ (**b**), in the mean fibre direction along path 1 for *Growth*-${\mathscr {B}}_{0}$ (dash-dot) and *Growth*-${\mathscr {B}}_{t}$ (solid), at weeks 1, 3, and 5. The corresponding experimental measurement of $e_{ff}$ from a canine heart mid-anterior wall by Costa et al. (1997) is also plotted in (**a**) for comparison

We then compare the Eulerian residual strain and stress components, $e_{ff}$ and $\tau _{ff}$, along the mean fibre direction, calculated from the residually stressed configuration ${\mathscr {B}}_{\tau _t}$ as shown in Fig. 4. The transmural distributions of $e_{ff}$ and $\tau _{ff}$ at weeks 0 to 5 are shown in Fig. 7 for both models. We can see that the trends of these distributions are opposite in the two models. For *Growth*-${\mathscr {B}}_{0}$, $e_{ff}$ is positive from the endocardial surface, and decreases transmurally; but for *Growth*-${\mathscr {B}}_{t}$, $e_{ff}$ is negative from the endocardial surface, and increases transmurally. The trend of *Growth*-${\mathscr {B}}_{t}$ is supported by the published experiments and previous studies, e.g. Costa et al. (1997); Omens et al. (1993); Wang et al. (2014). In particular, the experimental data by Costa et al. (1997) measured from the mid-anterior part of the canine LV are drawn in Fig. 7 to illustrate the qualitative dis/agreements with the two models.

**Fig. 8:**
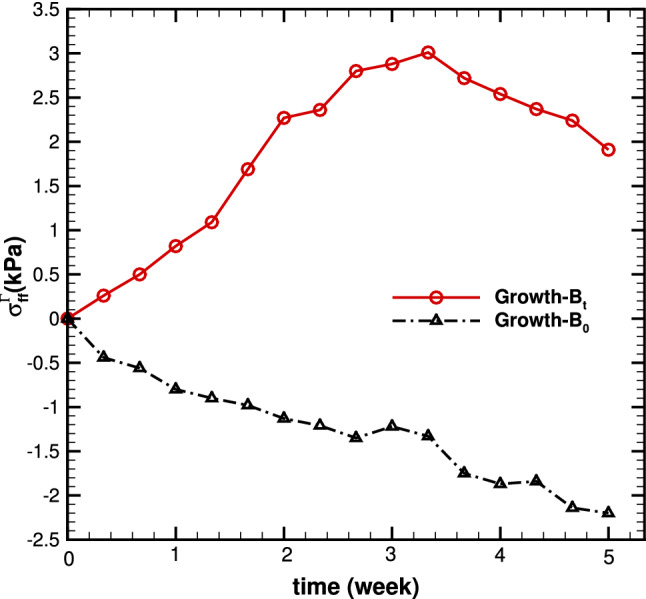
The time history of the maximum residual hoop stress $\tau _{ff}$, at growth time *t*=1-5 weeks in case 1 for *Growth*-${\mathscr {B}}_{t}$ (red solid with circle) and *Growth*-${\mathscr {B}}_{0}$ (black dash-dot with triangle)

#### LV case 2: Pathologic cardiac dilation

In the second example, we consider dilated cardiac growth, which is often a result of myocardial infarction (heart attack). In this case, the heart responds to a volume overload while attempts to maintain the cardiac output at a physiological level (Cheng et al. 2006).

This cardiac dilation can be described as a strain-driven, transversely isotropic, and irreversible growth. Again we follow Göktepe et al. (2010a) and model the process assuming

$$\begin{aligned} \vartheta ^s = \vartheta ^g \quad \text {and} \quad \vartheta ^s= \vartheta ^n \equiv 1. \end{aligned}$$

For *Growth*-${\mathscr {B}}_{0}$, the growth tensor is defined in ${\mathscr {B}}_{0}$ as

42 $$\begin{aligned} \mathbf{F }_{g}= \mathbf{I} + ({\vartheta }-1) \mathbf{f} _0 \otimes \mathbf{f} _0. \end{aligned}$$

The strain-driven involution law is Göktepe et al. (2010a),

43 $$\begin{aligned} {\dot{\vartheta }}= l (\vartheta ) \phi (\lambda _{f_0}), \end{aligned}$$

where $l(\vartheta )$ is the same as in (41)$_1$, $\lambda _{f_0}$ is the stretch along the direction of $\mathbf{f} _0$, i.e. $\lambda _{f_0}=( \mathbf{f} _0 \cdot \mathbf{C} \mathbf{f} _0)^{1/2}$, and $\phi$ obeys

44 $$\begin{aligned} \phi = \lambda _{f_0} - \lambda _{f_0}^{crit}, \end{aligned}$$

where $\lambda ^{crit}_{f_0}$ is the criteria value of elastic stretch. The parameters are chosen to be

45 $$\begin{aligned} \lambda ^{crit}_{f_0}=1.001, \quad \vartheta ^{max} = 1.5, \quad \gamma =2, \quad \beta =1, \end{aligned}$$

following Göktepe et al. (2010a).

For *Growth*-${\mathscr {B}}_{t}$, we have the incremental growth tensor defined in ${\mathscr {B}}_{t}$,

46 $$\begin{aligned} \bar{\mathbf{F}}_{g_t}/dt= \mathbf{I} + ({\dot{\vartheta }}-1) \mathbf{f} _t \otimes \mathbf{f} _t. \end{aligned}$$

We use the same strain-driven law as (43), except the function $\phi$ is function of $\lambda _{f}=( \mathbf{f} _t \cdot \mathbf{C} _{E_t} \mathbf{f} _t)^{1/2}$,

47 $$\begin{aligned} \phi (\lambda _{f})= \lambda _{f} - \lambda _{f}^{crit}, \end{aligned}$$

where $\lambda ^{crit}_{f}$ is the criteria value of elastic stretch along $\mathbf{f} _t$. In this case, the fibre structure in either ${\mathscr {B}}_t$ or ${\mathscr {B}}_g$ changes with time, so we need to compute the updated fibre structures using (32) and (36). Other than this, we keep the same parameters as in (45) with $\lambda ^{crit}_{f}$ replacing $\lambda ^{crit}_{f_0}$.

**Fig. 9:**
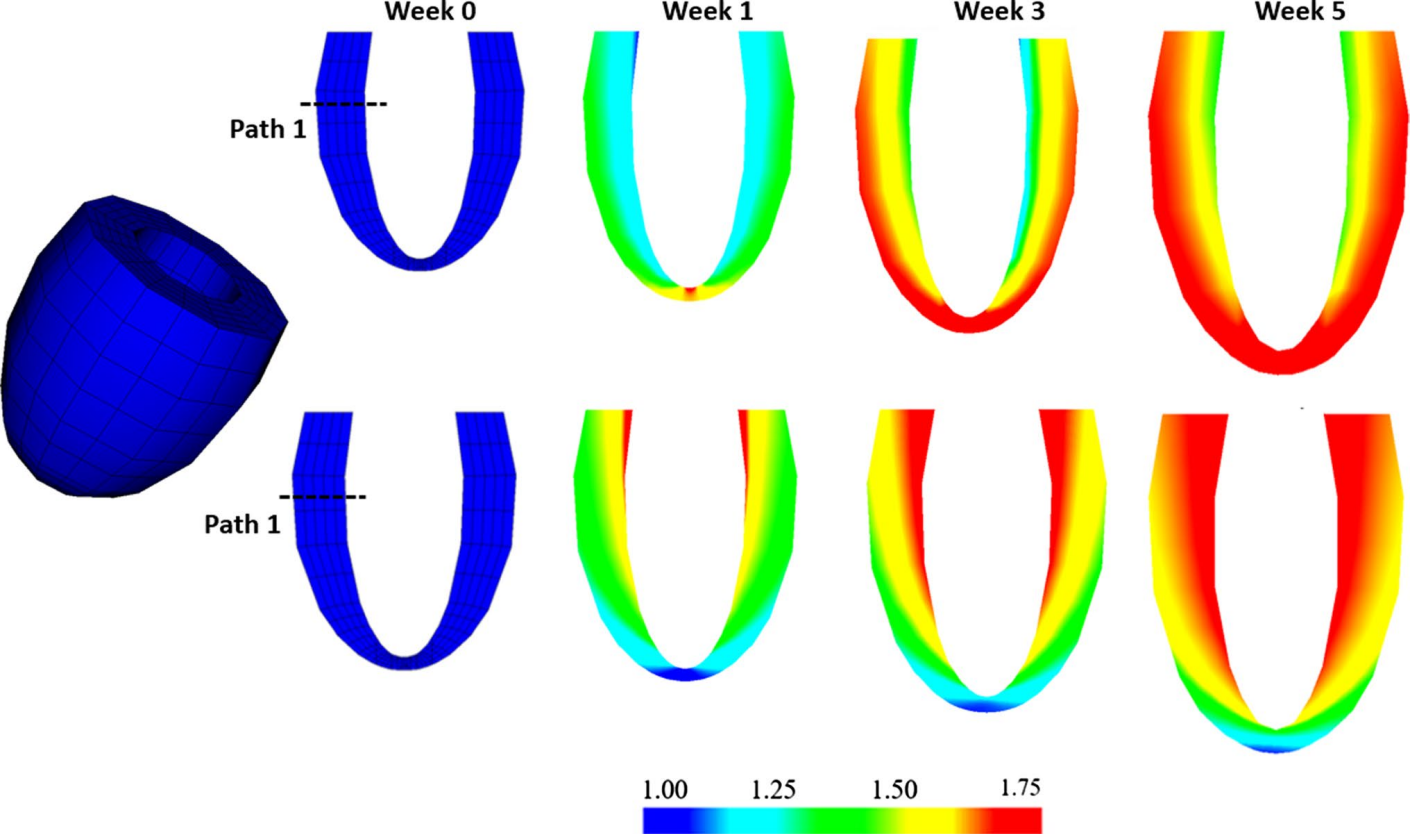
Contours of the growth factor $\vartheta$ in the Case 2 after 0, 1, 3, and 5 weeks growth. Above: Growth-${\mathscr {B}}_0$. Below: Growth-${\mathscr {B}}_t$. In both cases, the LV cavity size increases more obviously than the wall thickness

The distribution of $\vartheta$ for both models is compared in Fig. 9. Again, similar patterns are seen in each model, with more growth in the apex area for *Growth*-${\mathscr {B}}_{0}$, and more growth away from the apex area for *Growth*-${\mathscr {B}}_{t}$. We also see that compared to the isotropic growth in the previous example, in both models, this strain-driven cardiac growth (eccentric dilation) shows a more significant increase in the cavity size, but with a less noticeable increase in the wall thickness.

**Fig. 10:**
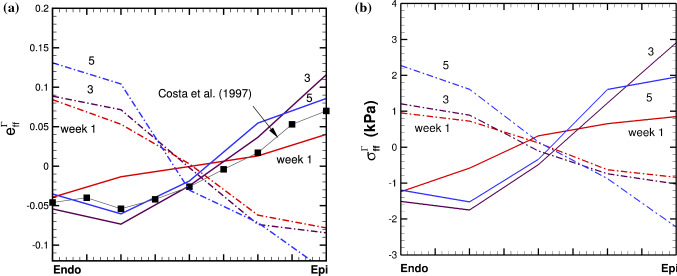
As in Fig. 7, but for the Example 2

The residual strain $e_{ff}$ and stress $\tau _{ff}$ along the Path 1 of the LV are shown in Fig. 10 for both models. Again, the two models have opposite strain distributions. Note that we do not see wall thinning of the LV wall in either model, which is frequently observed in clinic cases, e.g. (Hankiewicz et al. 2008; Venco et al. 1987), or modelled in (Zhuan et al. 2019). However, here no local growth or infarction zone is considered, which is responsible for wall thinning. Similar simulation and conclusion are seen in (Göktepe et al. 2010b; Lee et al. 2015; Klepach et al. 2012).

Similar trends of the maximum residual loop stress in Fig. 8 are observed in this case.

### Saturated growth in a multi-layer cylinder

To understand the differences between the two approaches in greater depth, here we consider a stress-driven growth in a cylindrical model subject to internal pressure, similar to that studied by (Goriely et al. 2008), except we use four layers here to enable the cross-layer growth. This model also allows us to compare to the special case considered by (Goriely et al. 2008), where a constant growth tensor (saturated homogenized growth) is used.

The model geometry in terms of the polar coordinates (*R*, $\varTheta$, *Z*) is given by

48 $$\begin{aligned}&R_1^{(\mathrm {i})} \leqslant R \leqslant R_1^{(\mathrm {o})}, \quad 0 \leqslant \varTheta \leqslant 2\pi , \quad 0\leqslant Z \leqslant L \quad \mathrm {(layer}\ I), \end{aligned}$$

49 $$\begin{aligned}&R_1^{(\mathrm {o})} \leqslant R \leqslant R_2^{(\mathrm {o})}, \quad 0 \leqslant \varTheta \leqslant 2\pi , \quad 0\leqslant Z \leqslant L \quad \mathrm {(layer}\ II), \end{aligned}$$

50 $$\begin{aligned}&R_2^{(\mathrm {o})} \leqslant R \leqslant R_3^{(\mathrm {o})}, \quad 0 \leqslant \varTheta \leqslant 2\pi , \quad 0\leqslant Z \leqslant L \quad \mathrm {(layer}\ III),\end{aligned}$$

51 $$\begin{aligned}&R_3^{(\mathrm {o})} \leqslant R \leqslant R_4^{(\mathrm {o})}, \quad 0 \leqslant \varTheta \leqslant 2\pi , \quad 0\leqslant Z \leqslant L \quad \mathrm {(layer}\ IIII), \end{aligned}$$

where *L* is the LV length, and $R_j^{(\mathrm {i})}$, $R_j^{(\mathrm {o})}$ denote the inner and outer radii of each layer ($j=1,2,3, 4$), respectively.

In terms of cylindrical polar coordinates (*r*, $\theta$, *z*), the geometry of the deformed configuration is given by

52 $$\begin{aligned} r=r (R), \quad \theta = \varTheta ,\quad z=\lambda Z, \end{aligned}$$

where $\lambda$ is the axial stretch. Similar to the incremental growth used by Goriely et al. (2008), we choose the incremental growth tensor in $Growth\text{- }{\mathscr {B}}_{t}$ to be

53 $$\begin{aligned} \bar{\mathbf{F}}_{g_t} = \text {diag} (1, d\vartheta , 1) , \end{aligned}$$

and the growth tensor for $Growth\text{- }{\mathscr {B}}_{0}$ to be

54 $$\begin{aligned} \mathbf{F} _{g}=\text {diag} (1, \vartheta , 1), \end{aligned}$$

with a simplified stress-driven growth involution law as in (Genet et al. 2015),

55 $$\begin{aligned} {\dot{\vartheta }}= \frac{1}{\beta } (\frac{\vartheta ^{\text {max}} -\vartheta }{\vartheta ^{\text {max}} -1}) \text {tr}(\varvec{\sigma }) , \quad \text {for } Growth\text{- }{\mathscr {B}}_{t}, \end{aligned}$$

and

56 $$\begin{aligned} {\dot{\vartheta }}= \frac{1}{\beta } (\frac{\vartheta ^{\text {max}} -\vartheta }{\vartheta ^{\text {max}} -1}) \text {tr}(\mathbf{M} ) , \quad \text {for } Growth\text{- }{\mathscr {B}}_{0}, \end{aligned}$$

where $\beta$ and $\vartheta ^{\text {max}}$ are chosen to be 1 and 1.75, which are arbitrarily set to reach the limit (saturated growth) sooner. Notice for this simple model, the rotation tensor caused by elastic deformation is always identity; therefore, the cumulative growth tensor $\mathbf{F}_{G_t}$ of $Growth\text{- }{\mathscr {B}}_{t}$ is the same as the growth tensor $\mathbf{F} _{g}$ in $Growth\text{- }{\mathscr {B}}_{0}$.

Note in the approach by Goriely et al. (2008), no stress- or strain- driven law is used; the cumulative growth tensor for the saturated growth is simply given as

57 $$\begin{aligned} \mathbf{F} _{G_t}=\text {diag} (1, \vartheta ^{\infty }, 1), \end{aligned}$$

where $\vartheta ^{\infty }$ is chosen to be $\vartheta ^{\text {max}}$ here.

**Fig. 11:**
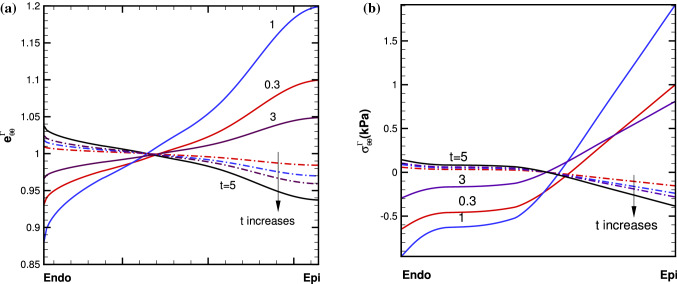
Transmural distributions of **a** residual hoop strain $e^{\varGamma }_{\theta \theta }$, and **b** residual hoop stress $\sigma ^{\varGamma }_{\theta \theta }$, at growth time *t* =0.3, 1, 3, 5, for the 4-layer cylindrical model using *Growth*-${\mathscr {B}}_{t}$ (solid) and *Growth*-${\mathscr {B}}_{0}$ (dash-dot) approaches. Both models reach the same limit as time goes to infinite ($t > 5$), when an uniform growth is reached everywhere

**Fig. 12:**
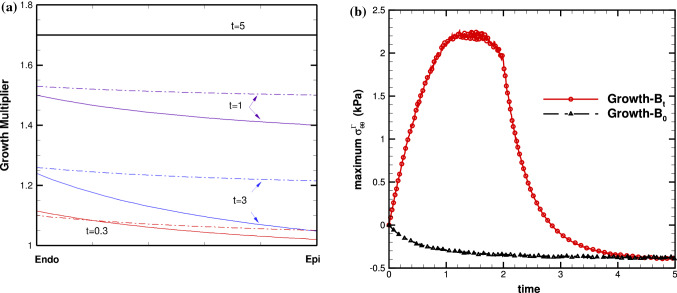
Transmural distributions of **a** the growth factor $\vartheta$ for *Growth*-${\mathscr {B}}_{t}$ (solid) and *Growth*-${\mathscr {B}}_{0}$ (dash-dot), (b) time history of the maximum residual hoop stress $\sigma ^{\varGamma }_{ff}$ for *Growth*-${\mathscr {B}}_{t}$ (red solid with circle) and *Growth*-${\mathscr {B}}_{0}$ (black dash-dot with triangle), at growth time *t*=0.3, 1, 3, 5, for the 4-layer cylindrical model

Solving the momentum equilibrium equation with compatibility conditions as in (Zhuan and Luo 2020), we obtain solutions of the problem. Figure 11 shows the transmural distributions of the residual hoop strain $e^{\varGamma }_{\theta \theta }$ and stress $\sigma ^{\varGamma }_{\theta \theta }$ for the 4-layer cylindrical model at different growth times, using *Growth*-${\mathscr {B}}_{0}$ and *Growth*-${\mathscr {B}}_{t}$ approaches. Figure 12 plots the cumulative growth factor and the time history of the maximum value of $\sigma ^{\varGamma }_{\theta \theta }$ (the history of the residual loop strain has a similar trend, not shown).

Note that in both models, $\vartheta$ increases monotonically with time. However, the trend of stress/strain is different; it increases monotonically with time for *Growth*-${\mathscr {B}}_{0}$, whereas for *Growth*-${\mathscr {B}}_{t}$ it reaches a local maximum first before decreasing. When growth is saturated, *Growth*-${\mathscr {B}}_{0}$ and *Growth*-${\mathscr {B}}_{t}$ both reach the exactly same solution as in (Goriely et al. 2008).

To be more general, we can assume the cylinder is made of many layers of equal thickness *d* in the reference configuration. We consider the cumulative diagonal growth tensors for all the layers

58 $$\begin{aligned}&\mathbf{F}_{G_t}^j=\mathbf{F}_g^j=\text {diag}(1,\vartheta _j,1), \quad j=1,\nonumber \\&\quad 2, 3, ...\text {(from inner to outer walls)}. \end{aligned}$$

After the growth, the *j*th layer is deformed with the middle perimeter $2\pi (R_j^{(i)}+d/2) \vartheta _j,$ and the radius $(R_j^{(i)}+d/2) \vartheta _j$, where $R_j^{(i)}$ indicates the inner radius of the *j*th layer. Similarly, the middle radius of the next layer is $(R_{j+1}^{(i)}+d/2) \vartheta _{j+1},$ where $R_{j+1}^{(i)}$ indicates the inner radius of the $(j+1)$th layer. Material continuity (compatibility) requires that

59 $$\begin{aligned} (R_{j}^{(i)}+d/2) \vartheta _j+d=(R_{j+1}^{(i)}+d/2) \vartheta _{j+1}. \end{aligned}$$

Since $d=R_{j+1}^{(i)}-R_j^{(i)}$, (59) can be rearranged to read

60 $$\begin{aligned} \frac{\vartheta _{j+1}-\vartheta _j}{R_{j+1}^{(i)}-R_j^{(i)}} = \frac{\vartheta _{j+1}-1}{R_j^{(i)} }. \end{aligned}$$

If we consider infinitely many layers, i.e. taking the limit of $d \rightarrow$ 0, then (60) is simply

61 $$\begin{aligned} \vartheta '=\frac{1-\vartheta }{R}, \end{aligned}$$

where $\vartheta '$ is the derivative of with respect to *R*. This is the material compatibility condition. If (61) holds, then the local volumetric growth is compatible and does not induce residual stress. If (61) is true everywhere, then the whole configuration is residually stress free. Hence, we can introduce the measure of incompatibility as

$$\begin{aligned} G(R,t)=\frac{1-\vartheta }{R}-\vartheta ' \end{aligned}$$

If $G > 0$, the growth will induce a smaller residual hoop stress in the *j*th layer compared with the $(j+1)$th layer. If $G > 0$ across the whole wall, then the hoop stress distribution will be from negative (compression) to positive (stretching). Likewise, if $G < 0$, the growth will induce a greater residual hoop stress in the *j*th layer compared with the $(j+1)$th layer. If $G < 0$ across the whole wall, then the hoop stress distribution will be positive to negative.

Since the cylindrical model is subject to internal pressure, the distribution of the Cauchy hoop stress is from positive to negative in the absence of growth. With the given growth (58), we found $G>0$ across the whole wall initially in the $Growth-{\mathscr {B}}_{t}$ model, leading to a positive maximum residual hoop stress towards the outer-most wall, as shown in Fig. 7b. This is opposite to the distribution of the Cauchy hoop stress, and tends to even out the overall stress distribution. In other words, the residual stress should have a beneficial effect under physiological loading. As this trend continues, growth, driven by the total stress, will have to slow down at some stage, and the growth mismatch, hence, the residual stress, will start to reduce. This occurs at t=1.5 in Fig. 12.

On the other hand, in the $Growth-{\mathscr {B}}_{0}$ approach, we found $G<0$ across the whole wall initially, leading to the negative maximum hoop stress towards the outer-most wall, as shown in Fig. 7a. This distribution of the residual loop stress holds the same trend with the loading induced Cauchy stress. Hence, the overall stress increases, which in turn enlarges the growth mismatch. The (negative) residual hoop stress becomes more negative with time.

Since growth is set to be limited by $\vartheta ^\text {max}$, giving sufficient time, all the material points eventually reach the same saturated growth in both approaches, so we have

$$\begin{aligned} \vartheta =\vartheta ^\text {max} > 1, \ \vartheta ' =0, \end{aligned}$$

which leads to $$ G <0$$ everywhere. In other words, for a saturated growth, we have the same negative maximum hoop stress using both approaches, as predicted by Goriely et al. (2008).

## Discussion

In this paper, we have developed a new approach that allows the growth of soft tissues to occur in the current loaded and residually stressed configuration, which is different to the traditional approaches that need to define growth tensor from the reference (unloaded and zero-stressed) configuration. Since all functional activities and remodelling processes in living tissues continuously occur and respond to bi-environmental signals, it is natural to assume that growth is induced in the current configuration to adapt to the time-dependent changes. Although we also assumed that the growth tensor is diagonal in the examples, our theory also works for more general expressions of the growth tensor.

The striking differences in the current approach, *Growth*-${\mathscr {B}}_{t}$, and the previous approach, *Growth*-${\mathscr {B}}_{0}$, are demonstrated in two test cases. Namely, isotropic growth and pathologic dilation of myocardium. The most significant finding is that the estimated residual strain distributions post G&R have opposite transmural distributions using the two approaches. And the result of the *Growth*-${\mathscr {B}}_{t}$ model is supported by experimental observations. The other differences we noticed are that in the *Growth*-${\mathscr {B}}_{t}$ model, the overall residual fibre stress or strain seems to reach a peak first (e.g. about week 3), and then decreases with time. This is different to the *Growth*-${\mathscr {B}}_{0}$ approach, where the residual stress or strain increase monotonically with time until the set growth limit is reached, as shown in Fig. 8.

These differences are explained in detail using a cylindrical model. Indeed, the cylindrical model has the same characteristics as the myocardium models, in terms of the behaviours of the two approaches. Further, with this model, we are able to introduce an incompatibility index, which can be used to judge the trend of the transmural distribution of residual stress. It is also easier to demonstrate that at sufficiently long time ($$t> 5 $$ in this case), both models converge to the same saturated growth limit (black curve), as the growth factor of all regions reaches the set limit of $$\vartheta ^{\text {max}}$$, which is the same solution of the constant growth studied by Goriely et al. (2008) for $$\vartheta > 1$$.

For the saturated growth in the cylindrical model, we have the same negative maximum hoop stress using both approaches, as predicted by Goriely et al. (2008). In other words, in the saturated growth, the residual stress does not help the soft tissue cope with the external loading, which is against the experimental observations. However, we remark that this limiting steady state is a result of the prescribed growth law, which may never be achieved in physical situations within a realistic time scale. Not only because the residual hoop stress distribution of the limiting case is not seen in experiments, but geometrical and time constraints of a living tissue/organ may not allow a homogenized (saturated) growth to occur. Clearly, in the two myocardium cases, none has reached the saturated growth.

We remark that the cumulative volumetric growth $\mathbf{F} _{G_t}$ from ${\mathscr {B}}_{0}$ essentially plays the same role as the conventionally used growth tensor $\mathbf{F} _g^0$ in (2). In other words, once we know $\mathbf{F} _{G_t}$, all the classical theories of G&R follow. However, it is impossible to define an evolution law for $\mathbf{F} _{G_t}$ prior to deformation, since elements of $\mathbf{F} _{G_t}$ are continuously rotated due to elastic deformation, as shown in (26) and (27). On the other hand, one can easily prescribe an evolution law for the incremental growth tensor $\mathbf{F} _{g_t}$. This explains why the updated growth theory by Goriely and Amar (2007) works for symmetrical geometries with diagonal deformation gradient and growth tensors, since deformation induced rotations do not make any differences. In this special case, a growth law for $\mathbf{F} _{G_t}$ may be defined prior to deformation.

To be more specific, we now show that under the assumption that both the deformation gradient and growth tensors are diagonal, the residual stress tensor arising from the incremental growth tensor ${\bar{\mathbf {F}}}_{gk}$ used by Goriely and Amar (2007)(i.e. “$\mathbf{R}_2\mathbf{A}_1$” in their Fig.2), is the same as in our approach. Note the rotation of ${\bar{\mathbf {F}}}_{gk}$ is an identity tensor due to its diagonality (i.e. “ ${\mathbf{R}_2}''\equiv {\mathbf{I}}$)) and “${\mathbf{A}_1}$” is essentially the mapping from ${\mathscr {B}}_{g_k}$ to ${{\bar{B}}}_{g_k}$ in Fig. 4, using our notation this is ${\mathbf{A}_k} {\mathbf{F}_{G_{k-1}} ^{-1}}$. The connection of this mapping to ${\bar{\mathbf{F }}}_{gk}$ is simply ${\bar{\mathbf {F}}}_{gk} \mathbf{A}_k {\bar{\mathbf{F }}}_{gk} ^{-1}$. Since all the tensors are diagonal and orders can be swapped, we can show that

$$\begin{aligned} {\bar{\mathbf {F}}}_{gk} \mathbf{A}_k {\bar{\mathbf {F}}}_{gk} ^{-1} ={\bar{\mathbf {F}}}_{gk} \mathbf{A}_k (\mathbf{R}_{\tau k} {\bar{\mathbf{F }}}_{gk}{} \mathbf{R}_{\tau k}^T\mathbf{F}_{G_{k-1}})^{-1} =\mathbf{A}_k (\mathbf{R}_{\tau k}{} \mathbf{R}_{\tau k}^T\mathbf{F}_{G_{k-1}})^{-1} =\mathbf{A}_k \mathbf{F}_{G_{k-1}} ^{-1}. \end{aligned}$$

In other words, for this special case, we obtain the same results as in Goriely and Amar (2007). However, when either the growth tensor or the elastic tensor is not diagonal, these two approaches are different. Hence, for general G&R processes, our residual stress tensor ${\bar{\mathbf {F}}}_{gk} \mathbf{A}_k{\bar{\mathbf {F}}}_{gk} ^{-1}$ cannot be simplified to $\mathbf{A}_k \mathbf{F}_{G_{k-1}} ^{-1}$ and is dependent on the past loading history.

G&R modelling is useful even if we do not know the history of the tissue growth. Often we encounter situations when all we know is that residual stress exists. A growth theory can be used to establishes the relationship between the residual strain/stress and the history of tissue growth. Hence, with a given growth tensor, we can estimate the residual strain (Zhuan and Luo 2020). On the other hand, by estimating the residual strain experimentally, e.g. via the opening angle methods, we can determine the cumulative growth tensor at the time when the residual stress is released. By considering growth in the current configuration, we also naturally included the effects of changed fibre orientations in the soft tissue. Indeed, the mean angle of the fibre distributions changes after continuous growth, which in turn updates the strain energy function.

Finally, we would like to remark that in this work a simplified HO model is used and the tuning factors in the test cases are not based on experiments. However, we believe these do not affect the qualitative behaviour of the outcome. Although our focus is on the myocardium tissue, the developed G&R framework is applicable to all soft tissues, and could pave the way for more realistic growth modelling. In reality, the limit of tissue growth should be dictated by local maximum stress/strain or physiological homeostasis, given boundary conditions. It is seldom that a biological organ can reach a saturated global growth. We remark that finding a suitable growth limit based on experimental evidence is crucially important for practical applications. This research topic remains a challenge.

## Conclusion

In this paper, we proposed a new theory of volumetric growth which depends on fields evaluated in the current configuration. In contrast to most previous models that have to define soft tissue growth in the natural configuration, we allow such a growth to be evaluated in the deformed and loaded configurations, without imposing any geometrical restrictions. We illustrated our idea using a simplified left ventricle model, that admits inhomogeneous growth in residually stressed and loaded configurations. We then compared our residual stress distribution with a typical previous volumetric growth model in which the growth tensor is defined in the natural configuration. Our results show that the new framework leads to a qualitative agreement with experiments, in contrast to the results from the previous model. In addition, using a multi-layer cylindrical model, we explained the nature of the differences in these two approaches using an incompatibility index, and demonstrated why, after an infinitely long time, both approaches attain the same homogenized/saturated growth state as identified by Goriely et al. (2008). We further observed that this limit may never be reached in soft tissue growth, since a state of saturated growth is unlikely to occur due to geometric and time constraints in a living organ. In addition, the distribution of the saturated residual stress increases the overall stress, and therefore acts contrary to the well-known observation that the residual stress reduces the stress in the wall under external loading. Perhaps it is for this reason, that the residual loop stress distribution at the saturation limit has not been observed in experiments on arteries or the heart. We also proved that our theory agrees with that of  Goriely and Amar (2007) under the same assumptions of symmetric growth and deformation. However, for general elastic deformation, the updated growth tensor using our approach becomes a function of elastic deformation from all previous loadings. Given the wide range of growth and remodelling processes in the living tissues, our theory is an important step forward in studying the time-responses of live organs to external factors soft tissues.

## Appendix: Thermodynamics compatibility

We now demonstrate that our constitutive approach for soft tissue growth satisfies the second law of thermodynamics. In other words, the system has a non-decreasing entropy 1.

We consider the constitutive law (5) and (28) that includes the growth, but we drop the index *k* and the incompressibility constrain to be more general. For an elastic tissue with growth, the mass density $\rho _0 (\mathbf{X} , t)$ in the reference configuration ${\mathscr {B}}_0$ changes with time, and can be computed from the push back of the mass density $\rho (\mathbf{x} , t)$ in the current configuration ${\mathscr {B}}_t$, i.e.

62 $$\begin{aligned} \rho _0 (\mathbf{X} , t) = J \rho (\mathbf{x} , t), \end{aligned}$$

where $J=\det (\mathbf{A} ^t)$. Time derivative of $\rho _0 (\mathbf{X} , t)$ gives

63 $$\begin{aligned}&{\dot{\rho }}_0 ={\dot{J}} \rho + J{\dot{\rho }}=J \rho \text {div}(\mathbf{v} ) + J (\frac{\partial \rho }{\partial t}+\mathbf{v} \cdot \text {grad} \rho )\nonumber \\&\quad =J(\frac{\partial \rho }{\partial t}+\text {div}(\rho \mathbf{v} )= J (r-\text {div}{} \mathbf{m} ), \end{aligned}$$

where *r* is the mass source per unit volume, $\mathbf{v}$ is the velocity vector and $\mathbf{m}$ is the mass flux across the boundary $\partial {\mathscr {B}}_t$.

Growth (or atrophy) is the result of mass production (or removal) and comes from a volumetric “source” *r* per unit volume in ${\mathscr {B}}_t$ and the boundary influx **m**, together written

$$\begin{aligned} \int _{{\mathscr {B}}_t} rdv -\int _{\partial {\mathscr {B}}_t} \mathbf{m} \cdot \mathbf{n} da, \end{aligned}$$

where $\mathbf{n}$ is the outward normal of ${\partial {\mathscr {B}}_t}$. The linear momentum balance equation for the growing tissue then becomes

64 $$\begin{aligned} \rho \dot{\mathbf{v }}= \rho \mathbf{b} ^*+\text {div}\varvec{\sigma }^* \end{aligned}$$

where $\rho \mathbf{b} ^*=\rho \mathbf{b} +(\text {div} \mathbf{m} ) \mathbf{v}$, $\varvec{\sigma }^*=\varvec{\sigma }-\mathbf{m} \otimes \mathbf{v}$. Let $k= \frac{1}{2} \mathbf{v} \cdot \mathbf{v}$ be the kinetic energy per unit mass, the total kinetic energy of the system is

$$\begin{aligned} K=\frac{1}{2} \int _{{\mathscr {B}}_t} \rho \mathbf{v} \cdot \mathbf{v} dv = \int _{{\mathscr {B}}_t} \rho k dv =\int _{{\mathscr {B}}_0} \rho _0 k dV, \end{aligned}$$

and the rate of K is

65 $$\begin{aligned} \frac{d K}{d t}=\int _{{\mathscr {B}}_0} ({\dot{\rho }}_0 k + \rho _0 {\dot{k}}) dV= \int _{{\mathscr {B}}_t} \rho \mathbf{v} \cdot \dot{\mathbf{v }} dv+\int _{{\mathscr {B}}_0} k{\dot{\rho }}_0dV. \end{aligned}$$

From the linear momentum equation (64) and the divergence theorem, we write the rate of force working $P^*$ as

66 $$\begin{aligned} \begin{aligned} P^*&=\int _{{\mathscr {B}}_t} \rho \mathbf{b} ^* \cdot \mathbf{v} dv + \int _{\partial {\mathscr {B}}_t} (\varvec{\sigma }^{*T} \mathbf{n} ) \cdot \mathbf{v} da \\&=\frac{d K}{d t} + \int _{{\mathscr {B}}_t} \text {tr}(\varvec{\sigma }^{*T} \mathbf{L} ) dv - \int _{{\mathscr {B}}_0} k {\dot{\rho }}_0 dV \end{aligned} \end{aligned}$$

where $\mathbf{L} =\nabla \mathbf{v}$ is the velocity gradient.

The energy balance of the system including thermal effects is now

67 $$\begin{aligned} \frac{d}{dt} (U+K) = P^*+Q+G \end{aligned}$$

where $U=\int _{{\mathscr {B}}_t} \rho udv$ is the total internal energy, with *u* being the internal energy of the body per unit mass, *G* is the rate of energy supply to feed the growth (or the additional entropy production term), and *Q* is the rate of heat production defined as

68 $$\begin{aligned} Q= \int _{{\mathscr {B}}_t} \rho h dv -\int _{\partial {\mathscr {B}}_t} \mathbf{q} \cdot \mathbf{n} da = \int _{{\mathscr {B}}_t} (\rho h - \text {div} \mathbf{q} ) dv, \end{aligned}$$

in which *h* is heart source per unit mass and **q** is the heat flux per unit area. (67) is then

69 $$\begin{aligned}&\int _{{\mathscr {B}}_t} \rho {\dot{u}} dv = \int _{{\mathscr {B}}_t} \text {tr}(\varvec{\sigma }^{*T} \mathbf{L} ) dv \nonumber \\&\quad +\int _{{\mathscr {B}}_t} (\rho h - \text {div} \mathbf{q} ) +G -\int _{{\mathscr {B}}_0} (k+u) {\dot{\rho }}_0 dV. \end{aligned}$$

We now take

$$\begin{aligned} G = \int _{{\mathscr {B}}_0} (k+u) {\dot{\rho }}_0 dV, \end{aligned}$$

so that last two terms cancel (and this not the only option, but is the simplest). We could think of *G* as consisting of bulk growth energy supply *g* (per unit mass) and energy flux $\mathbf{g}$ (per unit area): thus,

$$\begin{aligned} G=\int _{{\mathscr {B}}_t} \rho g dv - \int _{\partial {\mathscr {B}}_t} \mathbf{g} \cdot \mathbf{n} da =\int _{{\mathscr {B}}_t}(\rho g - \text {div}{} \mathbf{g} )dv, \end{aligned}$$

so locally we would have

$$\begin{aligned} \rho g - \text {div}{} \mathbf{g} =J^{-1}(k+u)\rho _0. \end{aligned}$$

Epstein and Maugin (2000) included in their energy balance a flux term, which is essentially $(k+u)\mathbf{m}$, that we have not included here, but again this does not influence the construction of constitutive laws unless one brings in second-gradient effects (which considerably complex the theory). The same applies to the various dissipative terms they include in the energy balance and entropy production.

The local form of the energy balance (69) can now be expressed as

70 $$\begin{aligned} \rho {\dot{u}} = \text {tr}(\varvec{\sigma }^{*T} \mathbf{L} ) +\rho h - \text {div} \mathbf{q} . \end{aligned}$$

Next define the free energy $\psi$ per unit mass by

$$\begin{aligned} \psi = u -T\eta , \end{aligned}$$

where $T > 0$ is the absolute temperature, and $\eta$ is the entropy per unit mass. Then, since the free energy is a function of density $\rho _0$, temperature *T* and deformation tensor, we can write (70) as

71 $$\begin{aligned}&(\rho \frac{\partial \psi }{\partial \mathbf{A} }: \dot{\mathbf{A ^t}}+ \rho \frac{\partial \psi }{\partial T} {\dot{T}} + \rho \frac{\partial \psi }{\partial \rho _0 } \dot{\rho _0} ) \nonumber \\&\quad + (\rho {\dot{T}} \eta + \rho T {\dot{\eta }}) = \text {tr}(\varvec{\sigma }^{*T} \mathbf{L} ) +\rho h - \text {div} \mathbf{q} . \end{aligned}$$

With this we associate the entropy equation—in global form this is

72 $$\begin{aligned} \frac{d}{dt}\int _{{\mathscr {B}}_t}\rho \eta dv = \int _{{\mathscr {B}}_t} \frac{\rho h}{T}dv-\int _{\partial {\mathscr {B}}_t}\frac{1}{T}{} \mathbf{q} \cdot \mathbf{n} da + \int _{{\mathscr {B}}_t}\rho {\mathscr {H}}dv, \end{aligned}$$

where ${\mathscr {H}}$ (a capital version of $\eta$) is the entropy production per unit mass, and in local form,

$$\begin{aligned} \rho {{\dot{\eta }}} - \frac{\rho h}{T} +\text {div} (\frac{\mathbf {q}}{T})= \rho {\mathscr {H}}. \end{aligned}$$

or

73 $$\begin{aligned} \rho T{\mathscr {H}}=\rho T{{\dot{\eta }}} - \rho h +\text {div} \mathbf{q} - \frac{1}{T}{} \mathbf{q} \cdot \text {grad}T. \end{aligned}$$

Combining this with (71), and assuming that the elastic material is isothermal (*T* is constant), we arrive at

74 $$\begin{aligned} \frac{\partial \psi }{\partial \rho _0 } \dot{\rho _0} = T{\mathscr {H}}, \end{aligned}$$

where we have made use of (5) and (28), following Ogden (1984), to get

75 $$\begin{aligned} \text {tr}(\varvec{\sigma }^* \mathbf{L} ) = J_{A^t}^{-1} \frac{\partial W_0}{\partial \mathbf{A} }: \dot{\mathbf{A }}^t= \rho \frac{\partial \psi }{\partial \mathbf{A} }: \dot{\mathbf{A }}^t. \end{aligned}$$

The second law of thermodynamics requires that ${\mathscr {H}} \ge 0$, this is known as the entropy production inequality (the entropy of an isolated system is non-decreasing—it evolves towards thermodynamics equilibrium). From (74), we can see that this is satisfied, as in general for a growing tissue we have

$$\begin{aligned} \quad \frac{\partial \psi }{\partial \rho _0 } \ge 0, \quad \text {and} \quad \dot{\rho _0} \ge 0. \end{aligned}$$

Thus, our constitutive modelling of the growing tissue is thermodynamically compatible as

76 $$\begin{aligned} {\mathscr {H}} \ge 0. \end{aligned}$$

On the other hand, if the living body is atrophic, i.e.

$$\begin{aligned} \quad \frac{\partial \psi }{\partial \rho _0 }< 0, \quad \text {and} \quad \dot{\rho _0} < 0, \end{aligned}$$

the entropy production inequality (76) still holds.
